# Radiomics Signature on Computed Tomography Imaging: Association With Lymph Node Metastasis in Patients With Gastric Cancer

**DOI:** 10.3389/fonc.2019.00340

**Published:** 2019-04-26

**Authors:** Yuming Jiang, Wei Wang, Chuanli Chen, Xiaodong Zhang, Xuefan Zha, Wenbing Lv, Jingjing Xie, Weicai Huang, Zepang Sun, Yanfeng Hu, Jiang Yu, Tuanjie Li, Zhiwei Zhou, Yikai Xu, Guoxin Li

**Affiliations:** ^1^Department of General Surgery, Nanfang Hospital, Southern Medical University, Guangzhou, China; ^2^Guangdong Key Laboratory of Liver Disease Research, The 3rd Affiliated Hospital of Sun Yat-sen University, Guangzhou, China; ^3^Department of Gastric Surgery, Sun Yat-sen University Cancer Center, Guangzhou, China; ^4^State Key Laboratory of Oncology in South China, Collaborative Innovation Center for Cancer Medicine, Guangzhou, China; ^5^Department of Medical Imaging Center, Nanfang Hospital, Southern Medical University, Guangzhou, China; ^6^Department of Radiology, The Third Affiliated Hospital of Southern Medical University, Guangzhou, China; ^7^School of Biomedical Engineering and Guangdong Provincal Key Laboratory of Medical Image Processing, Southern Medical University, Guangzhou, China; ^8^Center for Drug and Clinical Research, Nanfang Hospital, Southern Medical University, Guangzhou, China

**Keywords:** gastric cancer, lymph node metastasis, prediction, radiomics, nomogram

## Abstract

**Background:** To evaluate whether radiomic feature-based computed tomography (CT) imaging signatures allow prediction of lymph node (LN) metastasis in gastric cancer (GC) and to develop a preoperative nomogram for predicting LN status.

**Methods:** We retrospectively analyzed radiomics features of CT images in 1,689 consecutive patients from three cancer centers. The prediction model was developed in the training cohort and validated in internal and external validation cohorts. Lasso regression model was utilized to select features and build radiomics signature. Multivariable logistic regression analysis was utilized to develop the model. We integrated the radiomics signature, clinical T and N stage, and other independent clinicopathologic variables, and this was presented as a radiomics nomogram. The performance of the nomogram was assessed with calibration, discrimination, and clinical usefulness.

**Results:** The radiomics signature was significantly associated with pathological LN stage in training and validation cohorts. Multivariable logistic analysis found the radiomics signature was an independent predictor of LN metastasis. The nomogram showed good discrimination and calibration.

**Conclusions:** The newly developed radiomic signature was a powerful predictor of LN metastasis and the radiomics nomogram could facilitate the preoperative individualized prediction of LN status.

## Introduction

Gastric cancer (GC) is one of the most common malignant tumors and the second leading cause of cancer-related deaths worldwide (1). Accurate evaluation of lymph node metastasis (LNM) status in GC patients is vital for prognosis and treatment decisions (2–4). Some histopathologic factors and biomarkers (e.g., lymphatic invasion, matrix metalloproteinase-2) are found to be able to predict LNM in GC, but most of them are only available after surgery (5–9). Preoperative evaluation of LNM could provide meaningful messages for determining the options of adjuvant therapy and the adequacy of surgical resection, hence assisting in pretreatment decision making (2–4). D2 gastrectomy was accepted as the standard surgery, especially advanced GC (10). Recently, surgeons think of endoscopic resection as the best choice for early GC without LNM, on account of more postoperative complication and mortality of D2 gastrectomy (4). Besides, clinical node staging is often under estimating the higher node staging seen by pathology (3, 4). Therefore, accurate preoperative predictions of LNM status are vital for GC patients, especially at the early stage. Recent studies showed that several serum markers (e.g., serum human apurinic/apyrimidinic endonuclease 1, circulating microRNAs) could preoperatively predict LNM in GC, but these biomarkers still need further validation and are not a part of standard clinical practice (8).

Sentinel lymph nodes was proven to be effective in predicting LNM of breast cancer and malignant melanoma (11, 12). However, the drainage of the lymph node in GC is net-style, which is much more complicated than in breast cancer and melanoma. Thus, accurate location of the sentinel lymph node is very difficult in GC, even with nano tracers or 99mTc tin colloid (11, 13). Computed tomography (CT) scan, endoscopic ultrasonography (EUS), or PET-CT are currently commonly used to evaluate the preoperative staging of GC. The accuracy of these tools is still not satisfactory (13, 14).

Recent years, radiomics increasingly attracts attention, and it is the process of the conversion of imaging data into high dimensional mineable data via automatically extracting a large number of quantitative image features, followed by further data analysis for clinical decision support (15, 16). By combining multiple imaging features in parallel, radiomics enables the non-invasive profiling of tumor heterogeneity (15–19). Recent studies of radiomics have provided insights in personalized medicine in oncologic practice related to cancer detection, subtype classification, LNM, survival, and therapeutic response evaluation (15, 16, 18, 20–22). Although texture features of CT images have been reported to be related with survival in patients with GC (23–25), an optimal approach that integrates multiple imaging features as a predictive signature for LNM is quite necessary to be developed. However, there is still not a radiomics model that would enable excellent prediction of LNM in GC.

Hence, in the study, we want to develop a radiomics signature based on preoperative CT images to estimate the LNM in patients with GC and to further establish a radiomics nomogram that integrated the radiomics signature and clinicopathological findings for the individual preoperative prediction of LNM stage in GC patients.

## Materials and Methods

### Patients

The study enrolled three independent cohorts of 1,689 patients with GC. The training cohort and internal validation cohort that comprised 312 consecutive patients and 360 consecutive patients with total or partial radical gastrectomy were obtained from Nanfang Hospital of Southern Medical University (Guangzhou, China) between January 2007 and December 2013, January 2014, and December 2016, respectively. The external validation cohort comprising 1,017 consecutive patients was collected from Sun Yat-sen University Cancer Center and the third affiliated hospital of Southern Medical University between January 2008 and December 2012 with same enrollment criteria. Clinicopathological information was retrospectively collected for all these patients. The clinical sources of the 1,689 patients are listed in Table 1. All the patients satisfied the following inclusion criteria: histologically confirmed GC, standard unenhanced and contrast-enhanced abdominal CT performed <30 days before surgical resection, lymphadenectomy performed, and more than 15 lymph nodes harvested, complete clinicopathologic data, no combined malignant neoplasm, no distant metastasis, no preoperative chemotherapy. We excluded patients if the lesions of tumor could not be identified by CT or if they previously had received any anticancer therapy. Baseline clinicopathologic information, including age, gender, preoperative differentiation status, carcinoembryonic antigen (CEA), and cancer antigen 19-9 (CA19-9) was derived from medical records. Patients' clinical T stage (cT) and N stage (cN) and dates of CT imaging were also obtained. Ethical approval was obtained for this retrospective study at the three participating centers, and the informed consent requirement was waved.

**Table 1:** Descriptive statistics for gastric cancer cohorts.

**Variables**	**Training cohort (*****N*** **=** **312)**		**Internal validation cohort (*****N*** **=3 60)**		**External validation cohort (*****N*** **=** **1,017)**	
	***N***	**%**	***N***	**%**	***N***	**%**
**Age (Years)**						
≥60	119	38.1	154	42.8	405	39.8
<60	193	61.9	206	57.2	612	60.2
**Gender**						
Male	216	69.2	256	71.1	681	67
Female	96	30.8	104	28.9	336	33
**Size**						
≥4cm	168	53.8	163	47.9	634	62.3
<4cm	144	46.2	177	52.1	383	37.3
**Differentiation**						
Well	28	9	40	11.1	121	11.9
Moderate	93	29.8	100	27.8	181	17.8
Poor or undifferentiation	191	61.2	220	61.1	715	70.3
**Location**						
Cardia	64	20.5	81	22.5	276	27.1
Body	51	16.3	93	25.8	230	22.6
Antrum	154	49.4	168	46.7	452	44.4
Whole	43	13.8	18	5	59	5.8
**CEA**						
elevated	59	18.9	112	31.1	191	19.4
normal	253	81.1	268	68.9	826	80.6
**CA199**						
elevated	93	29.8	110	30.6	184	18.1
normal	219	70.2	250	69.4	833	81.9
**Clinical T stage**						
T1	14	4.5	30	8.3	129	12.7
T2	28	9	44	12.2	118	11.6
T3	39	12.5	68	18.9	291	28.6
T4a	191	61.2	183	50.8	324	31.9
T4b	40	12.8	35	9.7	155	15.2
**Clinical N stage**						
N0	97	31.1	146	40.6	379	37.3
N1	149	47.8	75	20.8	268	26.4
N2	50	16	85	23.6	178	17.5
N3	16	5.1	54	15	192	18.9
**pT stage**						
pT1	20	6.4	48	13.3	123	12.1
pT2	22	7.1	29	8.1	118	11.6
pT3	18	5.8	55	15.3	229	22.5
pT4a	176	56.4	165	45.8	463	45.5
pT4b	76	24.4	63	17.5	84	8.3
**pN stage**						
pN0	72	23.1	123	34.2	321	31.6
pN1	57	18.3	53	14.7	141	13.9
pN2	73	23.4	52	14.4	159	15.6
pN3	110	35.2	132	36.7	396	38.9

Based on the pathological N stage (pN) of the TNM staging, LNM was assigned to one of the four outcome categories: no lymph nodes metastasis (pN0 stage, reference category); 1–2 lymph nodes metastasis (pN1 stage); 3–6 lymph nodes metastasis (pN2 stage); or ≥7 lymph nodes metastasis (pN3 stage). Hence, the outcome of this study was multinomial.

#### Image Acquisition

All these patients underwent contrast-enhanced abdominal CT using the multidetector row CT (MDCT) systems (GE Lightspeed 16, GE Healthcare Milwaukee, WI; 64-section LightSpeed VCT, GE Medical Systems, Milwaukee, Wis; or 256-MDCT scanner Brilliance iCT, Philips Healthcare, Cleveland, OH, USA). The acquisition parameters are as follows: 120 kV; 150–190 mAs; 0.5- or 0.4-second rotation time; detector collimation: 8 × 2.5 mm or 64 × 0.625 mm; field of view, 350 × 350 mm; matrix, 512 × 512. After routine non-enhanced CT, arterial and portal venous-phase contrast-enhanced CT were performed after delays of 28 s and 60 s following intravenous administration of 90–100 ml of iodinated contrast material (Ultravist 370, Bayer Schering Pharma, Berlin, Germany) at a rate of 3.0 or 3.5 ml/s with a pump injector (Ulrich CT Plus 150, Ulrich Medical, Ulm, Germany). Contrast-enhanced CT was reconstructed with a thickness of 2.5 mm. Portal venous phase CT images (thickness: 2.5 mm) were retrieved from the picture archiving and communication system (PACS) (Carestream, Canada) for image feature extraction because of well-differentiation of the tumor tissue from the adjacent tissue.

#### Imaging Texture Analysis

We used the popular texture analysis software (MaZda 4.6), which was developed within COST (European Cooperation in the field of Scientific and Technical Research) projects B11 and B21, and which has been utilized for a large number of studies in the field, for all texture calculations (25, 26). For every lesion, a single region of interest (ROI) was constructed manually under the supervision of a senior board-certified radiologist, specializing in abdominal imaging, on the transverse image section that depicted the maximum lesion diameter. The free-form ROI covered the whole area of the lesion, as manifested by the thickened gastric wall. Gray-level normalization of each ROI was performed, using the limitation of dynamics to μ ± 3σ (μ, gray-level mean; and σ, gray-level standard deviation) to minimize the impact of contrast and brightness variation, which may otherwise overcurtain the true image texture (27). Besides, texture features derived from the gray-level histogram, the co-occurrence matrix, the run-length matrix, the absolute gradient, the autoregressive model, and the wavelet transform were calculated. The process of texture feature calculation only takes a few seconds per ROI. A detailed list of the individual texture features could be found in the MaZda documentation (26). The detailed list of all these features is shown in Supplementary Methods, Supplementary Table S10.

The inter-observer reproducibility was initially analyzed with 100 randomly chosen images for ROI-based texture feature extraction by two experienced radiologists (readers 1 and 2, with 11 and 10 years of clinical experience in abdominal CT study interpretation, respectively) (Figure S1). The complete details are shown in Supplementary Methods.

#### Radiomics Feature Selection and Signature Building

The LASSO logistic regression model was utilized to identify the optimal radiomics features for predicting pN stage from all these texture features, and then a multiple-feature-based radiomics signature, the radiomics score (Rad-score), was developed for predicting pN stage in the training cohort (28, 29). The LASSO regression was performed using R software version 3.4.0 with the “glmnet” package. Complete details are shown in the Supplementary Materials.

#### Development of an Individualized Prediction Model

Univariable associations between candidate predictors and the different outcome categories were estimated with multinomial logistic regression analysis. Multinomial logistic regression allows for simultaneous estimation of the probability of the different outcomes (pN1, pN2, pN3, and pN0 stage [the reference category]) (Figure S2) (30, 31). Essentially, the multinomial logistic regression model includes several logistic regression models simultaneously, to evaluate the relationship between the predictors and each of the outcomes compared with the reference category (30, 31). Therefore, estimated regression coefficients of the predictors might differ per outcome (32).

Multivariate multinomial logistic regression analysis was performed, which formed the basis for the pN stage prediction model. In the training cohort, univariate logistic regression analysis was performed for different variable values and variables that achieved statistical significance at *P* < 0.05 were entered into the multivariate analyses. Backward step-wise selection was applied by utilizing the likelihood ratio test with Akaike's information criterion as the stopping rule (30, 31). The model was also implemented into nomograms to enable use on plain paper and implementation as a calculation tool.

#### Validation of the Prediction Model

The prediction model was validated by measuring both discrimination and calibration. Both discrimination and calibration were evaluated by bootstrapping with 1,000 resamples. Discrimination was evaluated by the concordance index (C-index). The area under the receiver operating characteristic curve (AUC) was also used to measure discriminative ability of binary models representing each pathological N stage (pN1, pN2, pN3) compared to pN0 stage (33). Similarly, calibration plots were used to graphically display agreement between the predicted and actual probability of each pN stage, based on the binary models.

#### Clinical Use

Decision curve analysis (DCA) was conducted to evaluate the potential net benefit of the predictive models (34). The AUC value only shows the discriminative accuracy of a model (35). Whereas, DCA, which is a recently proposed novel method for evaluating predictive model, visualizes the clinical impacts of a treatment strategy (34, 36). This represents a potential net benefit of each decision strategy at each threshold probability. DCA was carried out to compare the clinical usefulness of the radiomics nomogram, Rad-score and cN stage by quantifying the net benefits at different threshold probabilities.

#### Statistical Analysis

Continuous variables are expressed as mean ± SD and compared using an unpaired, 2-tailed *t* test, one-way ANOVA or Mann-Whitney test. Categorical variables were compared using the χ2 test or Fisher exact test. Nomograms and calibration plots were generated using the rms package of R version 3.4.0 (*http://www.r-project.org*). C-index calculation was performed with the “Hmisc” package. All other statistical analyses were conducted using R version 3.4.0 and SPSS version 21.0 (IBM). All statistical tests were 2-sided and *P* < 0.05 was considered statistically significant.

## Results

### Clinical Characteristics

The clinicopathologic characteristics of patients in the training cohort (*n* = 312), internal (*n* = 360), and external validation cohort (*n* = 1,017) are listed in Table 1 and Tables S1–S3. And, the clinical characteristics of patients were similar among the three cohorts. In internal cohort (training and internal validation cohorts), pN1 stage occurred in 110 (16.4%) patients, pN2 in 125 (18.6%), pN3 in 242 (36.0%), and pN0 occurred in 195 (29.0%) patients. In external validation cohort, pN1 stage occurred in 141 (13.9%) patients, pN2 in 159 (15.6%), pN3 in 396 (38.9%), and pN0 occurred in 321 (31.6%) patients (Table 1).

The inter- and intra-observer reproducibility of the texture features extraction was high (Supplementary Material). Hence, all outcomes were based on the measurements of the first radiologist.

### Feature Selection and Radiomics Signature Development

Of the texture features, 269 features were reduced to 15 potential predictors on the basis of 312 patients in the training cohort (21:1 ratio; Figure S3), and were features with non-zero coefficients in the LASSO logistic regression model. The features were constructed as a radiomics signature, which was presented in the Rad-score calculation formula: **Rad-score** = 0.026593967 ^*^ Kurtosis + 0.035877138 ^*^ S(2, 0)Entropy + 1.278065286 ^*^ S(2, 2)InvDfMom + 0.195298398 ^*^ S(3, 3)Correlat + 0.138878969 ^*^ S(4, -4)Correlat + 0.000378754^*^ S(4, -4)SumVarnc - 0.000113878 ^*^ S(5, 5)Contrast + 0.087501674 ^*^ S(5, 5)Correlat - 0.00152959 ^*^ S(5, 5)SumOfSqs - 0.20114528 ^*^ S(5, 5)DifEntrp + 0.81106837 ^*^ S(5, -5)InvDfMom + 0.002575539 ^*^ Vertl_GLevNonU +−0.003948845 ^*^ WavEnHH_s-2 - 0.000270237 ^*^ WavEnHH_s-4 - 0.000242295 ^*^ WavEnHL_s-5. Tables S1–S3 showed the relationships between the Rad-score, clinicopathological characteristics, and pN stage in the training and validation cohorts.

We used three heat maps to determine the association between radiomics features, Rad-score and pN stage in each cohort (Figures S4–S6). The results showed significant positive correlation between Rad-score and pN stage in the training, internal and external validation cohort (correlation coefficient: 0.47, 0.41, 0.40; respectively, all *P* < 0.0001). Significant negative correlations were found between pN stage and signature features S (5, 5)Contrast, WavEnHH_s-2, WavEnHH_s-4, and WavEnHL_s-5. S(2, 2)InvDfMom, S(3, 3)Correlat, S(5, -5)InvDfMom, Vertl_GLevNonU were positively correlated with pN stage.

#### Diagnostic Validation of Radiomics Signature

**Figure 1:**
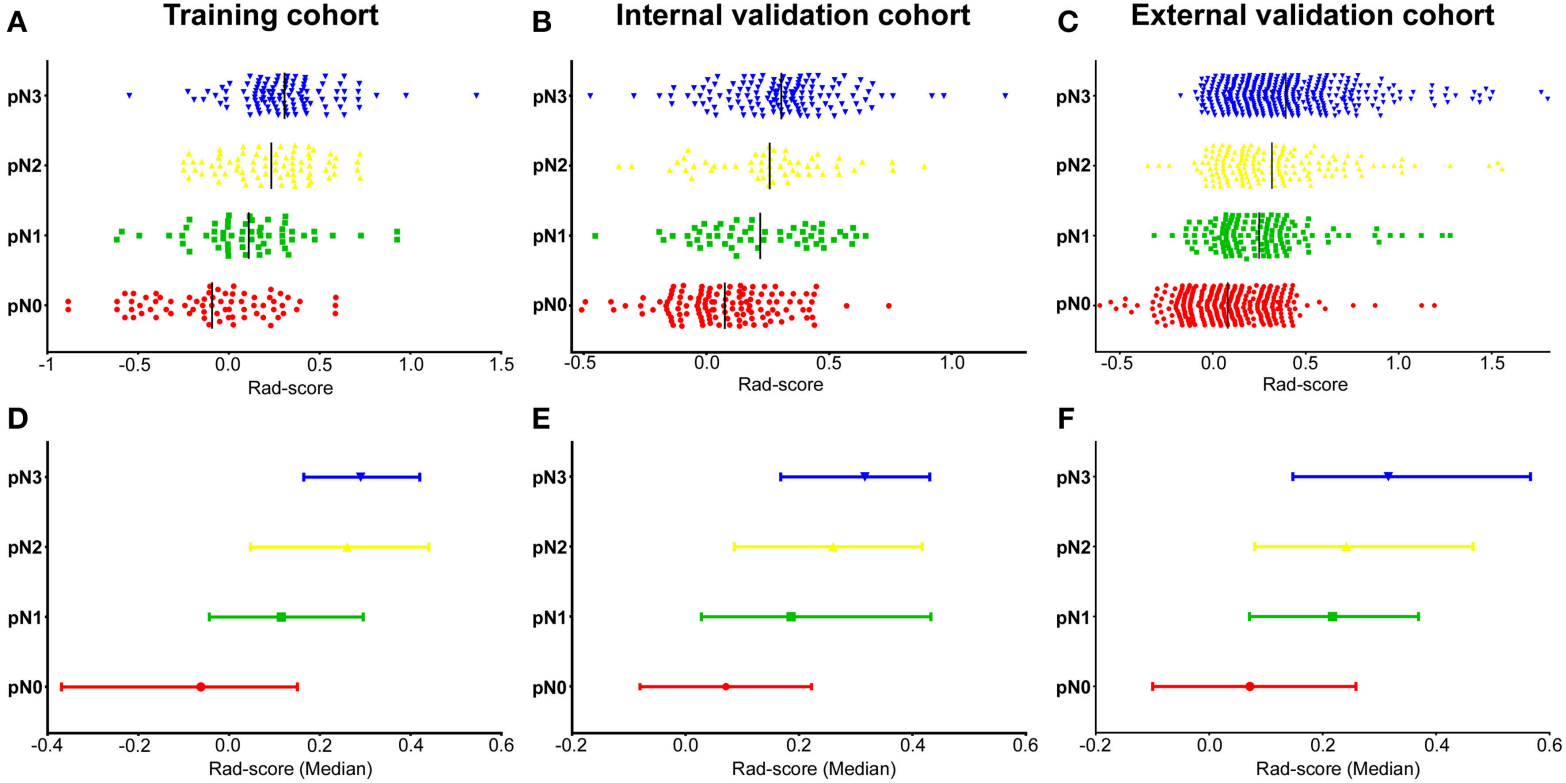
Distribution of radiomics scores regarding the classification of pN stage (pN1, pN2, pN3 vs. pN0) in the training, internal, and external validation cohorts. **(A–C)** Scatter plots of the radiomics scores (Rad-scores) in the training **(A)**, internal **(B)**, and external validation **(C)** cohorts; The black solid lines present mean values. **(D–F)** Represents the median values and 95% CI of radiomics scores distribution in the training **(D)**, internal **(E)**, and external validation **(F)** cohorts. pN, pathological N stage.

There was a significant difference in Rad-score between pN1, pN2, pN3, and pN0 patients in the training cohort (*P* < 0.001, Figure 1), which was confirmed in the internal and external validation cohorts (*P* < 0.001, Figure 1). Higher pN stage patients generally had higher Rad-scores in the training and validation cohorts. The radiomics signature yielded a C-index of 0.704 (95% CI, 0.661–0.744) in training cohort, and 0.674 (0.634–0.715) and 0.687 (0.662–0.711) in internal and external validation cohorts, respectively. When stratified analysis was performed according to clinicopathological risk factors, the Rad-score were still significantly associated with pN stage in the training, internal, and external validation cohorts (Tables S4–S6).

#### Development of an Individualized Prediction Model

**Figure 2:**
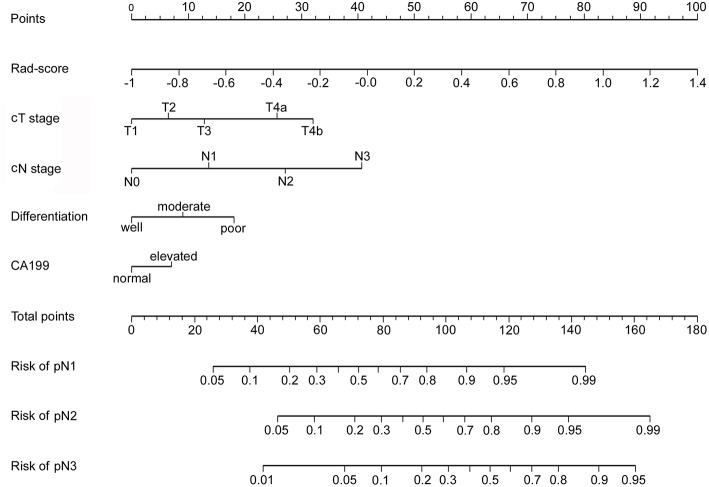
Developed radiomics nomogram. The radiomics nomogram was developed in the training cohort, with the radiomics signature, cT stage and cN stage, differentiation status, and CA199 level incorporated.

In univariable analysis, the radiomics signature were significantly associated with pN stage (Table S7). Variables demonstrating a significant effect were included in the multivariable analysis. Multivariate logistic regression analysis after adjustment for clinicopathological factors demonstrated that the radiomics signature remained a powerful and independent predictor for pN stage in the training, internal and external validation cohorts (Table 2). Then, we constructed a nomogram, integrating the radiomics signature, preoperative differentiation status, CA199 level, cT and cN stages, based on the coefficients of the multivariate analysis in the training cohort (Figure 2). The DCA curve of a nomogram maps the predicted probabilities into points on a scale from 0 to 100 in a user-friendly graphical interface. The total points accumulated by the various variables correspond to the predicted probability for a patient (22, 25, 37, 38). To use the nomogram for a patient, firstly draw a vertical line to the top points row to assign points for each variable; then, add the points of each variable together and drop a vertical line of the total points row to obtain the probability of pN1, pN2, pN3 stage for each patient (Figure 2). A calculating tool (Figure S7) is also implemented that could calculate the estimated the probability of pN1, pN2, pN3 stage after the user inputs the needed patient and tumor characteristics. For example, for a patient with Rad-score of −0.40 and CT reported T4aN1 tumor that is poorly differentiated as well as elevated levels of CA19-9, the model predicts that the probability of pN1, pN2, pN3 stage were 75, 44, 18.5%, respectively (Figure S7).

**Table 2:** Multivariate association of Rad-score, clinicopathological characteristics with Lymph node metastasis in the training, internal and external validation cohorts.

**Variables**	**pN1 vs. pN0**		**pN2 vs. pN0**		**pN3 vs. pN0**	
	**OR (95%CI)**	***P***	***OR (95%CI)***	***P***	***OR (95%CI)***	***P***
**TRAINING COHORT**						
**Rad-score**	**7.769 (1.971-30.626)**	**0.003**	**45.225 (10.11–202.37)**	**<0.0001**	**110.99 (23.40–526.35)**	**<0.0001**
Differentiation	2.014 (1.131–3.587)	0.017	2.711 (1.458–5.043)	0.002	2.423 (1.289–4.554)	0.006
CA199	2.778 (0.936–8.247)	0.066	3.360 (1.146–9.845)	0.027	3.414 (1.148–10.15)	0.027
cT stage	1.509 (1.134–2.009)	0.005	1.536 (1.157–2.040)	0.003	2.131 (1.545–2.946)	<0.0001
cN stage	2.647 (1.357–5.162)	0.004	2.982 (1.534–5.796)	0.001	5.586 (2.841–10.98)	<0.0001
**INTERNAL VALIDATION COHORT**						
**Rad-score**	**12.619 (2.699–58.99)**	**0.001**	**18.966 (3.50–102.84)**	**0.0006**	**50.925 (11.37–228.04)**	**<0.0001**
Differentiation	1.496 (0.908–2.467)	0.114	2.759 (1.455–5.233)	0.002	2.311 (1.389–3.847)	0.001
CA199	2.225 (0.944–5.243)	0.068	0.995 (0.388–2.548)	0.992	1.162 (0.515–2.624)	0.718
cT stage	1.505 (1.183–1.917)	0.001	2.315 (1.680–3.191)	<0.0001	2.071 (1.608–2.666)	<0.0001
cN stage	1.168 (0.772–1.769)	0.462	1.713 (1.132–2.592)	0.011	2.520 (1.745–3.638)	<0.0001
**EXTERNAL VALIDATION COHORT**						
**Rad-score**	**14.737 (6.035–35.99)**	**0.001**	**30.056 (12.34–73.20)**	**<0.0001**	**57.442 (24.20–136.36)**	**<0.0001**
Differentiation	1.654 (1.198–2.283)	0.002	2.152 (1.506–3.075)	<0.0001	3.365 (2.336–4.848)	<0.0001
CA199	4.330 (2.118–8.850)	<0.0001	4.562 (2.206–9.431)	<0.0001	5.525 (2.710–11.26)	<0.0001
CEA	1.214 (0.628–2.347)	0.564	2.897 (1.614–5.202)	<0.0001	3.060 (1.720–5.446)	<0.0001
cT stage	1.150 (1.014–1.306)	0.03	1.327 (1.163–1.516)	<0.0001	1.602 (1.407–1.824)	<0.0001
cN stage	1.251 (0.999–1.567)	0.051	1.783 (1.437–2.211)	<0.0001	3.582 (2.917–4.398)	<0.0001

*Bold values denotes Rad-score*.

#### Validation of the Nomogram

**Figure 3:**
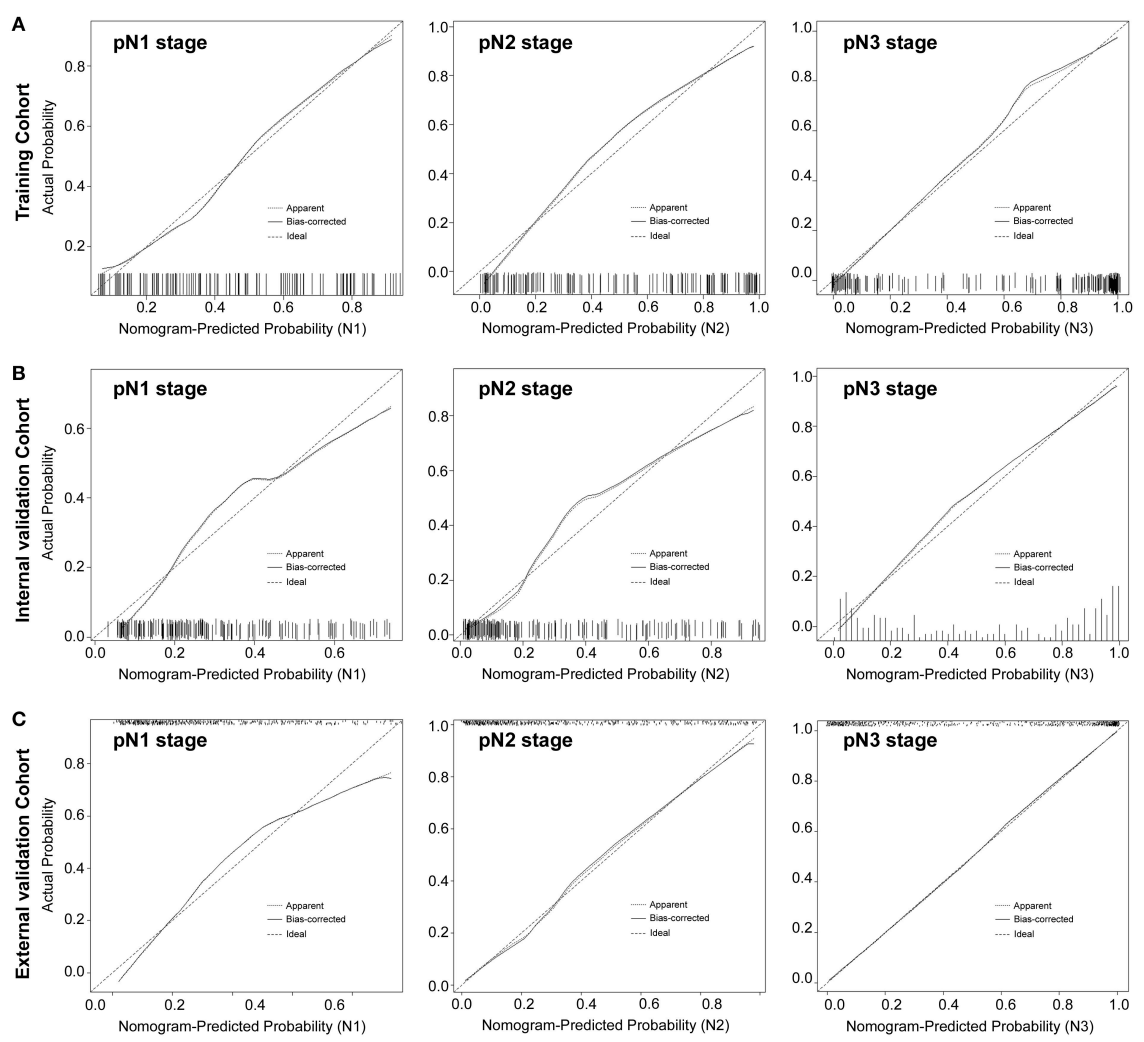
Calibration plots of each nomogram predicting pN1, pN2, pN3 stage. The calibration plot is a comparison between predicted and actual outcome. The 45-degree reference line represents an ideal model perfectly calibrated with an outcome. The solid line is the apparent accuracy of the nomogram, without correction for overfit. The dotted line is the bootstrap corrected performance of the nomogram, with a scatter estimate for future accuracy. pN1 stage, left panels; pN2 stage, middle panels; pN3 stage, right panels.

**Figure 4:**
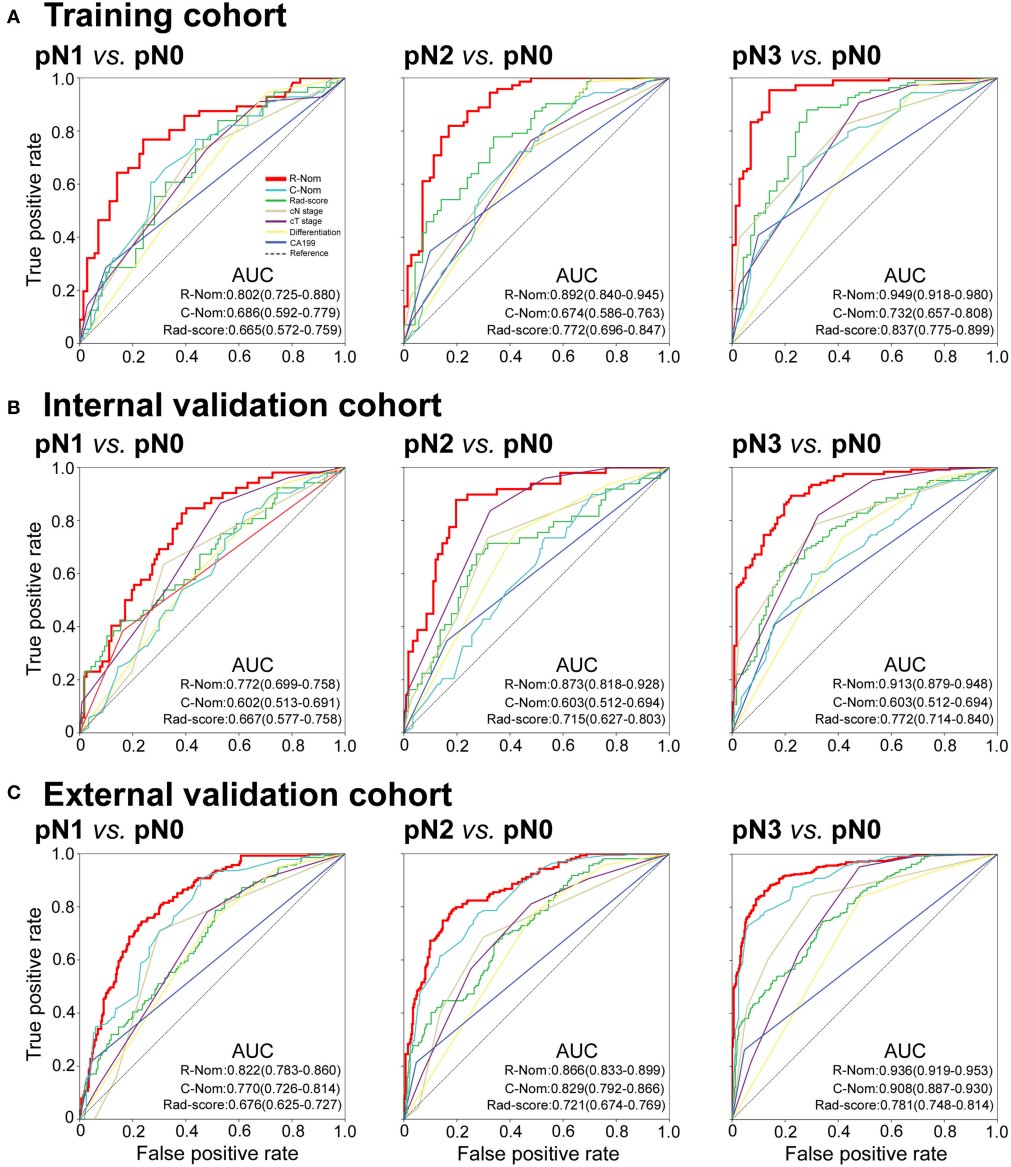
Receiver operating characteristic (ROC) curves of binary logistic regression models comparing pN1, pN2, or pN3 stage to pN0 stage in each cohort. pN1 vs. pN0: left panels; pN2 vs. pN0: middle panels; pN3 vs. pN0: right panels. R-Nom, Radiomics nomogram; C-Nom, Clinicopathological nomogram.

The calibration curve of the radiomics nomogram for the probability of pN1, pN2, pN3 stage revealed good agreement between prediction and observation in the training cohort (Figure 3A). The C-index for the nomogram was 0.788 (95% CI 0.752–0.825) for the training cohort. The model's AUCs for pN1, pN2, pN3 vs. pN0 were 0.802 (0.725–880), 0.892 (0.840–945), and 0.949 (0.918–980), respectively (Figure 4A and Table S8). And the model's AUCs were also calculated for pN2 vs. pN1, pN3 vs. pN1, and pN3 vs. pN2 (0.596 (0.496–0.695), 0.752 (0.673–0.832), and 0.683 (0.603–0.763), respectively; Figure S8A and Table S9).

Good calibration was also observed for the prediction of pN1, pN2, pN3 stage in the internal and external validation cohorts (Figures 3B,C). In the internal validation cohort, the model's C-index was 0.802 (0.769–0.836). The AUCs for the prediction of pN1, pN2, and pN3 were 0.772 (0.699–758), 0.873 (0.818–928), and 0.913 (0.879–948), respectively (Figure 4B). And the model's AUCs for pN2 vs. pN1, pN3 vs. pN1, and pN3 vs. pN2 were 0.664 (0.559–0.769), 0.750 (0.673–0.827), and 0.612 (0.524–0.701), respectively (Figure S8B and Table S9). In the external validation cohort, the model's C-index was 0.829 (0.810–0.847). The AUCs for pN1, pN2, and pN3 were 0.772 (0.699–758), 0.873 (0.818–928), and 0.913 (0.879–948), respectively (Figure 4C and Table S8). And the model's AUCs for pN2 vs. pN1, pN3 vs. pN1, and pN3 vs. pN2 were 0.627 (0.564–0.690), 0.796 (0.757–0.836), and 0.688 (0.641–0.735), respectively (Figure S8C and Table S9).

In addition, we also constructed the clinicopathological nomogram incorporating only the clinicopathological factors (preoperative differentiation status, CA199 level, cT, and cN stages) (Figure S9). And, the AUCs of the radiomics nomogram were higher than the AUCs of the clinicopathological factors for pN1, pN2, pN3 vs. pN0 in the training, internal, and external validation cohorts (Figure 4).

#### Clinical Use

**Figure 5:**
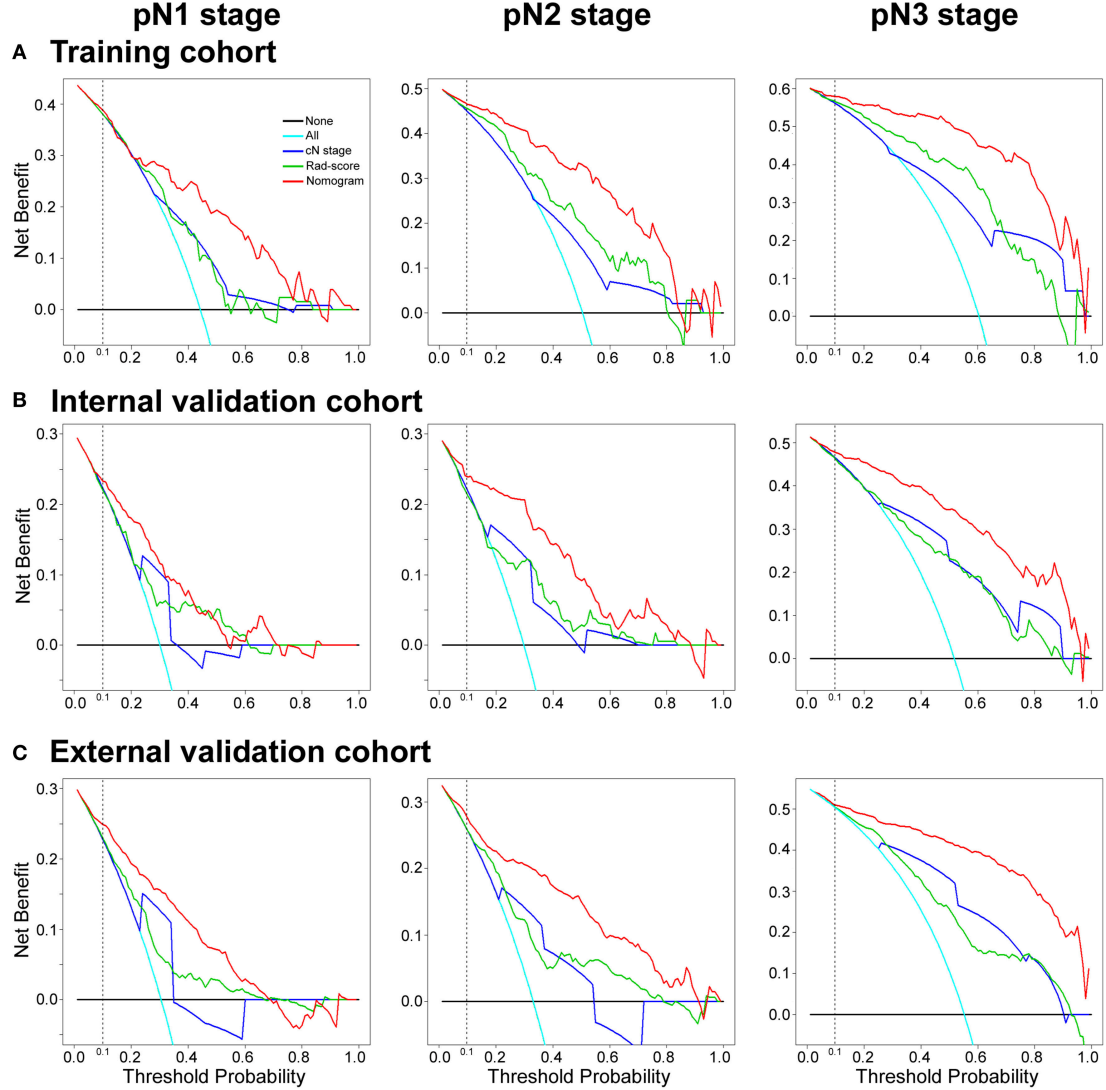
Decision curve analysis for the radiomics nomogram, Rad-score and Clinical N stage in the training, internal and external validation cohorts. The y-axis measures the net benefit. The red line represents the radiomics nomogram. The green line represents the radiomics score (Rad-score). The blue line represents the Clinical N stage (cN stage). The azure line represents the assumption that all patients were pN stage (pN1, pN2, or pN3 stage). Thin black line represents the assumption that no patients have LN metastases. The net benefit was calculated by subtracting the proportion of all patients who are false positives from the proportion who are true positives, weighting the relative harm of forgoing treatment against the negative consequences of an unnecessary treatment. Here, the relative harm was calculated by [pt/(1–pt)]. “pt” (threshold probability) is where the expected benefit of treatment is equal to the expected benefit of avoiding treatment; a patient considering treatment informs us how he or she weighs the relative harms of false positive results and false negative results ([a–c]/[b–d] = [1–pt]/pt); a–c is the harm from a false-negative result; b–d is the harm from a false-positive result. a, b, c, and d give the value of true positive, false positive, false negative, and true negative, respectively (34, 36). The decision curve showed that if the threshold probability of a patient or doctor is >10%, using the nomogram in the current study to predict pN stage adds more benefit than the treat-all-patients scheme or the treat-no-patients scheme.

The DCA curves of the nomogram in the training and validation cohorts were presented in Figure 5. The DCA curves showed that if the threshold probability of a physician or patient is >10%, using the nomogram to predict the pN stage provides more benefit than either the treat-no-patients scheme or the treat-all-patients scheme. The DCA showed that the integrated radiomics nomogram had a higher net benefit than the cN stage and Rad-score across the majority of the range of reasonable threshold probabilities in the training and validation cohorts (Figure 5).

## Discussion

In this study, we constructed a 15 texture features based radiomics signature that was significantly associated with LN metastasis and was an independent predictive factor of pN stage in patients with GC. Then, we constructed and validated a radiomics nomogram for the preoperative individualized prediction of pN stage. Incorporating the radiomics signature, preoperative differentiation status, CA199 level, cT, and cN stages, the easy-to-use nomogram may facilitate the preoperative individual prediction of LNM status.

The combined analysis of multiple markers as a signature, rather than individual analyses, is the approach that demonstrates the most promise to change clinical practice (20, 21, 28). The LASSO method is a popular mean for regression of high-dimensional variables (28, 29, 39). With the LASSO Cox regression model, we have built a five-immune feature signature that can predict disease-free survival and overall survival for patients with GC (40, 41). Furthermore, Jiang et al. developed and validated a 19 features radiomics signature of CT images that could predict GC survival and chemotherapeutic benefits (25). Besides, radiomics signature of Coroller et al. (18) F fluorodeoxyglucose PET images was also associated with survival and chemotherapeutic benefits in GC (22). For the construction of the radiomics signature in this study, 269 candidate radiomics features were cut down to 15 potential predictors by inspecting the predictor-outcome relationship by shrinking the regression coefficients with the LASSO regression. Similarly, the radiomics signature that integrated multiple individual imaging features demonstrated adequate discrimination CT scans of the abdomen are mandatory for precise preoperative T and N staging (4).

Radiomic studies on CT, PET, and MRI have reported that radiomics feature values vary through different image reconstruction, filtration, slice thickness, matrix size, exposure parameters, and type of scanners (42, 43). In the present study, the CT images were obtained from two scanners as many previous studies (20–22, 25), which was a limitation of this study. Thus, the variability of the values of radiomics features computed on CT images from different CT scanners should be considered, and the effects must be minimized in future studies of radiomics (42). Previous studies showed that first-order features were more reproducible than shape metrics and textural features (44). Entropy was consistently deemed as one of the most stable first-order features. There was still no emergent consensus with regard to either textural features or shape metrics; whereas, coarseness and contrast appeared among the least reproducible (44). Whereas, Lv et al. found that radiomics features depicting poor absolute-scale robustness regarding to parameter settings could still result in good diagnostic performance in nasopharyngeal PET/CT (45). In the same way, robustness of radiomics features ought not to be overemphasized for removal of features toward evaluation of clinical tasks (45). In this study, the radiomics signature, which was composed of 15 radiomics features, was significantly associated with pathological LN stage in the training cohort, which was also validated in the internal and external validation cohorts. Therefore, it is still necessary of devoted researches to select features with sufficient dynamic range among patients, with intra-patient reproducibility and low sensitivity to image acquisition and reconstruction protocols (46).

The accuracy of CT for the preoperative prediction of LN status was poor in GC patients (47, 48). Previous studies showed that the accuracy of pN stage by CT scan was only around 64–78%, even when these other techniques are used(47, 48); besides, the accuracy of EUS was 50–71.2% (49). PET-CT was also applied to preoperative identification of LNM and had advantages on distant LNM and bone metastasis (50). Nevertheless, the accuracy of PET-CT for regional LNM did not reveal an advantage over CT or EUS (50). Many studies have showed that several clinicopathological factors, for example depth of invasion, tumor size, differentiation type, CEA/CA199 level, lymphovascular invasion associated with LNM (2, 51, 52). Using these clinicopathological factors, several nomograms were established for prediction of LNM in early GC, but these nomograms still require further validation and no particular nomogram has been widely used in clinical practice (2, 51, 52). Recently, Huang et. al. presented a radiomics signature that could be useful for LNM prediction in colorectal cancer (21). Thus, we tried to develop an accurate model to preoperatively predict pN staging by combining radiomics features and the preoperative clinicopathological variables, including these tumor characteristics and serologic markers.

The standard treatment for advanced GC in East Asian countries is curative gastrectomy followed by postoperative chemotherapy, and the feasibility of utilizing neoadjuvant chemotherapy is currently being investigated (4, 53, 54). Several phase II or III clinical trials are ongoing to assess the efficacy of neoadjuvant chemotherapy in Japan. In these trials, GC patients with extensive LNM are receiving preoperative chemotherapy. Therefore, preoperative prediction of pN stage using the radiomics nomogram may help to screen patients who can benefit from neoadjuvant chemotherapy.

Gastrectomy with D2 dissection was a standard surgical procedure for resectable GC according to the treatment guidelines of the Japanese Gastric Cancer Association (JGCA) (4). Whereas, several studies from western countries showed that patients with GC treated by D2 dissection had a significantly higher rate of complications, a longer hospital stay and a higher postoperative mortality rate than those who had D1 dissection (4, 51). Thus, more attention should be paid to improve postoperative quality of life without impairing long-term survival. D2 gastrectomy seems to be an overly invasive surgery for pN0 patients. Hence, accurate preoperative predictions of LNM are crucial for patients, especially with early GC. Endoscopic mucosal resection and endoscopic submucosal dissection have been adopted as the least invasive procedures for the resection of early GC. In such circumstances, sentinel node (SN) concept has been introduced for early GC surgery. Recently, PINPOINT® (NOVADAQ, Canada) has been developed for indocyanine green (ICG) fluorescence guided surgery (55, 56). Ohdaira et al. showed a new method with ICG and PINPOINT® could facilitate the identification of ICG positive lymph nodes in SN mapping in back-table under room light, which may be able to be applied for avoiding for intraoperative SN mapping of laparoscopic GC surgery (55). Encouraged by several favorable single-institution reports, Kitagawa et al. conducted a multicenter, single-arm, phase II study of SN mapping that utilized a standardized dual tracer endoscopic injection technique with technetium 99 me labeled tin colloid and 1% isosulfan blue dye (57). Although several successful studies have been reported, there are still some controversial aspects as to the clinical application of SN mapping in GC, which has a relatively complicated lymphatic flow.

Our radiomics signature and nomogram could provide valuable information for preoperative prediction of pN stage. In the future, comprehensively considering the information of the radiomics features and the development of SN mapping, we may develop a preoperative prediction model for avoiding unnecessary D2 dissection, intra-operative SN mapping and modified laparoscopic surgery.

To provide a more individualized LNM prediction model, nomograms have been constructed to evaluate massive significant clinicopathologic predictors to better predict the outcomes of individual patients. Although, some nomograms were developed to predict the lymph node status for GC (2, 51, 52), no particular nomogram has been widely used in clinical practice. The previous nomograms only combined several clinicalpathological factors, and some models can't be used preoperatively (51), that lost the value of guide surgical operation. However, our radiomics nomogram incorporated the 15-feature radiomics signature and four preoperative clinical factors (preoperative differentiation status, preoperative CA199 level, and cT and cN stage), more comprehensively reflecting the status of the disease and obviously improving the accuracy. Validation of the nomogram was performed by calibration plots, the C-index and ROC analysis. The nomogram performed well with a good calibration and the C-index was satisfactory. Furthermore, our radiomics nomogram could preoperatively predict the pN stages with high AUCs both in internal and external cohorts (AUCs for pN1: 0.802 (95% CI 0.725–880), pN2: 0.892 (0.840–945), and pN3: 0.949 (0.918–980), respectively, in the training cohort), that could provide more valuable information to estimate the necessary of adjuvant therapy and the adequacy of surgical resection, thus assisting in pretreatment decision making.

According to the GC molecular classification of The Cancer Genome Atlas (TCGA), GC was classified into 4 subtypes: Epstein-Barr virus-positive, microsatellite instability, genomically stable, and chromosomal instability (58). Further understanding of GC molecular characterizations could give rise to new therapeutic strategies, which could contribute to understand the molecular mechanism of LNM. The association of GC molecular subtype and radiomics features was not clear, and should be explored in future studies. In oncology, radiogenomics represents a novel entity in clinical sciences that bidirectionally links imaging features with underlying molecular profile and thus could serve as a surrogate for noninvasive genomic correlation, prediction, and identification (19, 59). Banerjee et al. constructed a CT radiogenomic biomarker to predict microvascular invasion and clinical outcomes in hepatocellular carcinoma (19). On the basis of magnetic resonance image features, glioblastoma was divided into 3 distinct subtypes with distinct molecular pathway activities (59). In the future, we will dedicate to develop radiogenomic biomarkers for LNM prediction and treatment strategy decisions. Further studies in radiogenomics should devote to clarify the association between tumor genomics characteristics and their imaging appearance, and construct imaging features integrating phenotypic and genotypic metrics that could predict lymph node metastasis, recurrence risk or survival, and thus better stratify patients for more precise therapeutic care (60–62).

Whereas, there are some limitations of our study. The nomogram was developed and externally validated using three retrospective data sets from three Chinese institutions. The limitations of the study also included the uncertainty related to the measurement of radiomics features on the range of CT scanner used in the study. Besides, the use of contrast probably impacts the radiomics features and normalization may not be sufficient to overcome this influence. A multicenter, prospective study is needed to validate these results in a larger population in future. Moreover, other predictive biomarkers might be incorporated to improve the accuracy of the model.

In conclusion, our results showed the identified radiomics signature has the potential to be used as a biomarker for prediction of LN metastasis in patients with GC. In addition, our study showed that a radiomics nomogram that incorporated both the radiomics signature and clinicopathologic risk factors, could be conveniently applied to facilitate the preoperative individualized prediction of LN status in patients with GC.

## Author Contributions

Guarantor of the article**:** GL, YX, and ZZ. Conception and design: GL, YX, ZZ, YJ, TL, and XuZ. Collection and assembly of data: YJ, WW, CC, XiZ, YH, JX, JY, and WL. Data analysis and interpretation: YJ, WW, XiZ, JX, WH, ZS, and JY. Manuscript writing and final approval of manuscript: All authors.

### Conflict of Interest Statement

The authors declare that the research was conducted in the absence of any commercial or financial relationships that could be construed as a potential conflict of interest.
