# Supplementary material for: Radiomics Signature on Computed Tomography Imaging: Association With Lymph Node Metastasis in Patients With Gastric Cancer
Source: Front Oncol. 2019 Apr 26;9:340. doi: 10.3389/fonc.2019.00340 (PMC6498894; doi:10.3389/fonc.2019.00340)
Supplement: Supplementary file 1 [file Table_1.docx]

**Supplementary Materials**

1. **Supplementary Methods**
2. **Supplementary Reference**
3. **Supplementary Figures**
4. **Supplementary Tables**

**Supplementary Methods**

**Construction of Radiomics Signature using LASSO Logistic Regression Model**

The least absolute shrinkage and selection operator method (LASSO) is a popular method for regression of high-dimensional predictors.[^1-3^](#_ENREF_1) The method uses an L1 penalty to shrink some regression coefficients to exactly zero. We selected λ via 1-SE (standard error) criteria, i.e., the optimal λ is the largest value for which the partial likelihood deviance is within one SE of the smallest value of partial likelihood deviance. Thus, we plotted the partial likelihood deviance versus log (λ), where λ is the tuning parameter. A value λ = 0.099696 with log (λ) = -2.305632 was chosen by cross-validation via the 1-SE criteria. A vertical line was drawn at log (λ) = -2.305632, which corresponds to the optimal value λ = 0.099696 (**Figure S3**). The optimal tuning parameter resulted in fifteen non-zero coefficients. Fifteen features, Kurtosis, S(2,0)Entropy, S(2,2)InvDfMom, S(3,3)Correlat, S(4,-4)Correlat, S(4,-4)SumVarnc, S(5,5)Contrast, S(5,5)Correlat, S(5,5)SumOfSqs, S(5,5)DifEntrp, S(5,-5)InvDfMom, Vertl_GLevNonU, WavEnHH_s-2, WavEnHH_s-4, WavEnHL_s-5, with coefficients 0.026593967, 0.035877138, 1.278065286, 0.195298398, 0.138878969, 0.000378754, 0.000113878, 0.087501674, 0.00152959, 0.20114528, 0.81106837, 0.002575539, -0.003948845, 0.000270237, and 0.000242295, respectively, were selected in the LASSO logistic regression model (**Figure S3B**). At first, there were 279 texture features calculated from enhanced CT image of each patient, but missing values were observed in certain features derived from the autoregressive model (5 features: Teta1, Teta2, Teta3, Teta4, Sigma) and the absolute gradient (5 features: GrMean, GrVariance, GrSkewness, GrKurtosis, GrNonZeros) (**Table S10**). Therefore, we use all the remaining 269 texture features in the LASSO logistic regression model.

**Inter- and intra-observer Reproducibility of Feature Extraction**

The inter-observer and intra-observer reproducibility were initially analyzed with 100 randomly chosen images for ROI-based texture feature extraction by two experienced radiologists (readers 1 and 2, with 11 and 10 years of clinical experience in abdominal CT study interpretation, respectively). To assess the intra-observer reproducibility, reader 1 repeated the generation of texture features twice in a 4-week period following the same procedure. The workflow for the remaining images was completed by the first radiologist.

An independent samples t-test or Kruskal-Wallis H test, where appropriate, was used to assess the differences between the features generated by reader 1 (first time) and those by reader 2 as well as between the twice-generated features by reader 1. Inter- and intra-class correlation coefficients (ICCs) were used to evaluate the intra- and inter-observer agreement of features extraction. An ICC greater than 0.75 presents good agreement.

***Results*** Satisfactory inter- and intra-observer reproducibility of the texture feature extraction was achieved. There was no statistically significant difference between the features of the two readers “i.e.” between reader 1’s first-extracted features and those of the reader 2, with P values ranging from 0.61 to 0.86. The inter-observer ICCs of all metrics calculated on the basis of the “two” reader’s measurements were good, ranging from 0.75 to 0.94. The intra-observer ICC calculated based on reader 1’s twice feature extraction ranged from 0.79 to 0.95. Therefore, given the relatively heavy workload (1,689 patients), only the first radiologist drew all the patients’ CT images, and all outcomes were based on the measurement of the first reader.

**R Software Packages Used for Statistical Analysis**

LASSO logistic regression was performed using the “glmnet” package. Nomogram and calibration plots were done with the “rms” package. C-index calculation was performed the “Hmisc” package. Decision curve analysis was performed with the function of “dca.R”.

**Radiomics score (Rad-score) calculation formula:**

**Rad-score** = 0.026593967 * Kurtosis + 0.035877138 * S(2,0)Entropy + 1.278065286 * S(2,2)InvDfMom + 0.195298398 * S(3,3)Correlat + 0.138878969 * S(4,-4)Correlat + 0.000378754* S(4,-4)SumVarnc - 0.000113878 * S(5,5)Contrast + 0.087501674 * S(5,5)Correlat - 0.00152959 * S(5,5)SumOfSqs - 0.20114528 * S(5,5)DifEntrp + 0.81106837 * S(5,-5)InvDfMom + 0.002575539 * Vertl_GLevNonU + -0.003948845 * WavEnHH_s-2 - 0.000270237 * WavEnHH_s-4 - 0.000242295 * WavEnHL_s-5

**Supplementary Reference**

1. Jiang Y, Zhang Q, Hu Y, et al. ImmunoScore Signature: A Prognostic and Predictive Tool in Gastric Cancer. *Annals of surgery* 2018;267: 504-13.

2. Tibshirani R. The lasso method for variable selection in the Cox model. *Statistics in medicine* 1997;16: 385-95.

3. Tibshirani R. Regression shrinkage and selection via the lasso: a retrospective. *J R Stat Soc B* 2011;73: 273-82.


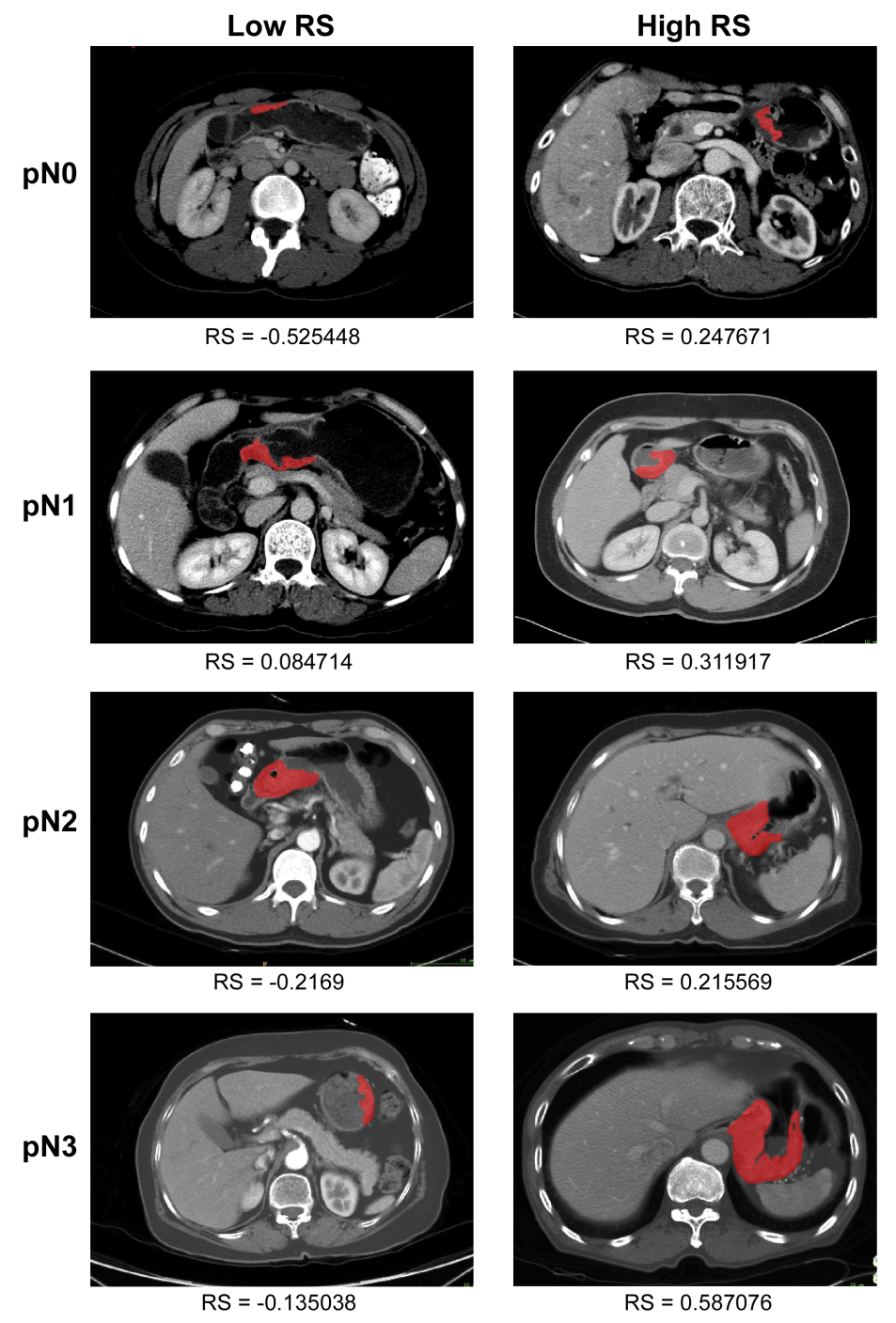


**Figure S1.** Representative images with region-of-interest (ROI) in different pN stage patients. Region-of-interest (ROI): red region. RS: Radiomics score.


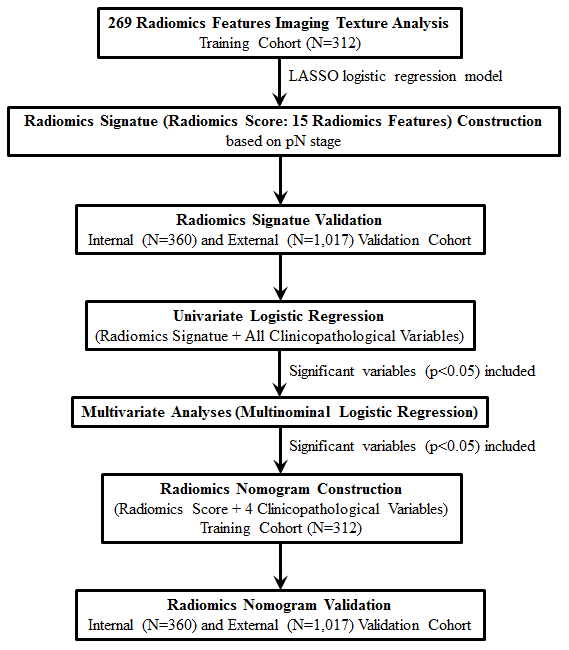


**Figure S2. Study design.**

**
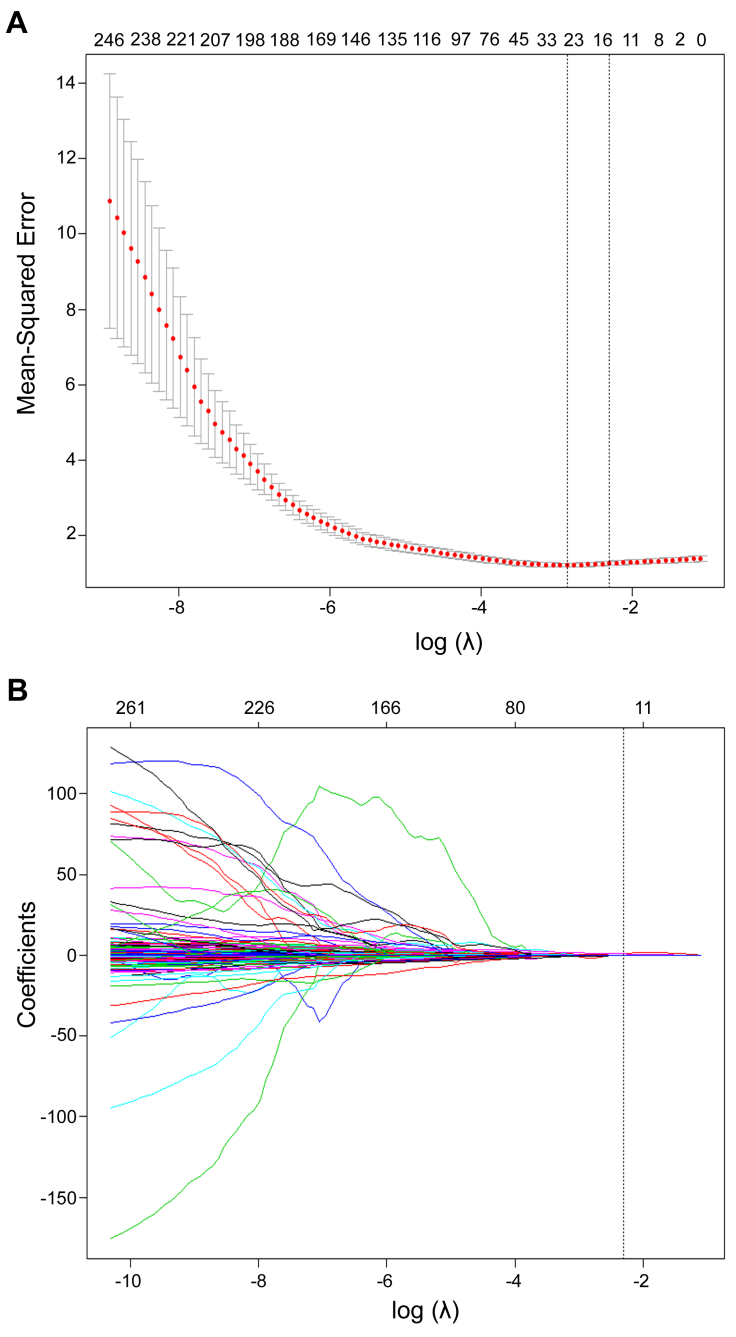
**

**Figure S3. Texture feature selection using the least absolute shrinkage and selection operator (LASSO) logistic regression model.** (A) Tuning parameter (λ) selection in the LASSO model used 10-fold cross-validation via minimum criteria. The partial likelihood deviance (PLD) curve was plotted versus log (λ). Dotted vertical lines were drawn at the optimal values by using the minimum criteria and 1 standard error of the minimum criteria (the 1-SE criteria). A λ value of 0.099696, with log (λ) of -2.305632 was chosen (1-SE criteria) according to 10-fold cross-validation. (B) LASSO coefficient profiles of the 269 texture features. A coefficient profile plot was produced against the log (λ) sequence. A vertical line was drawn at the value selected using 10-fold cross-validation, where optimal λ resulted in fifteen nonzero coefficients.


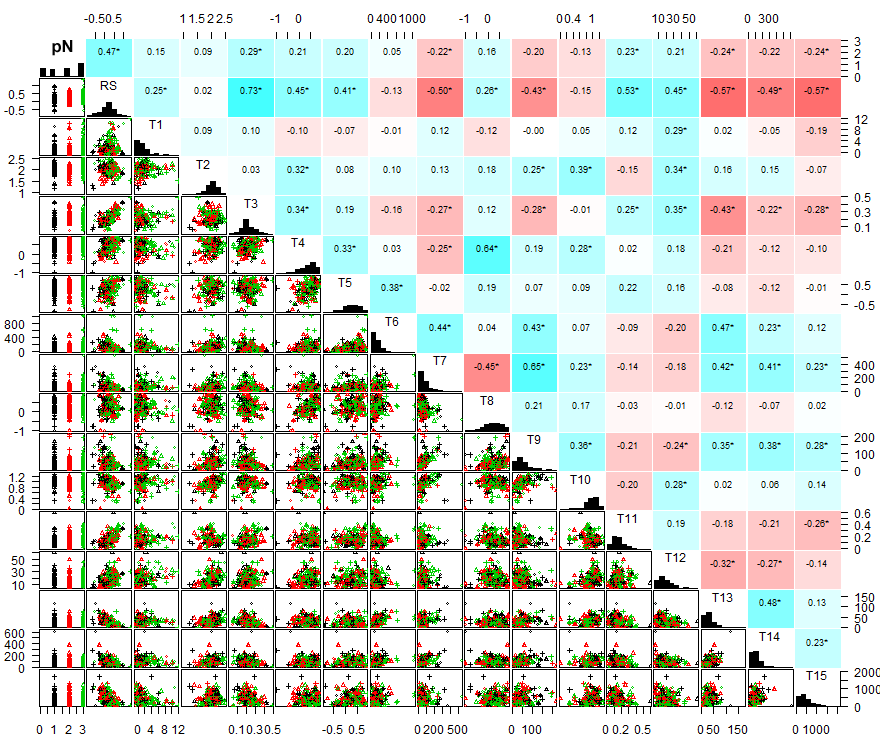


**Figure S4.** Scatterplot matrix of the interrelationship between the 15 radiomics features and Rad-score, pN stage in the training cohort. The values denote Pearson correlation coefficients, with numbers closer to 1 identifying a better correlation. *p<0.0001. pN: pathological N stage; RS: Rad-score; T1 to T15 represent the 15 radiomics features (Kurtosis, S(2,0)Entropy, S(2,2)InvDfMom, S(3,3)Correlat, S(4,-4)Correlat, S(4,-4)SumVarnc, S(5,5)Contrast, S(5,5)Correlat, S(5,5)SumOfSqs, S(5,5)DifEntrp, S(5,-5)InvDfMom, Vertl_GLevNonU, WavEnHH_s-2, WavEnHH_s-4, WavEnHL_s-5).


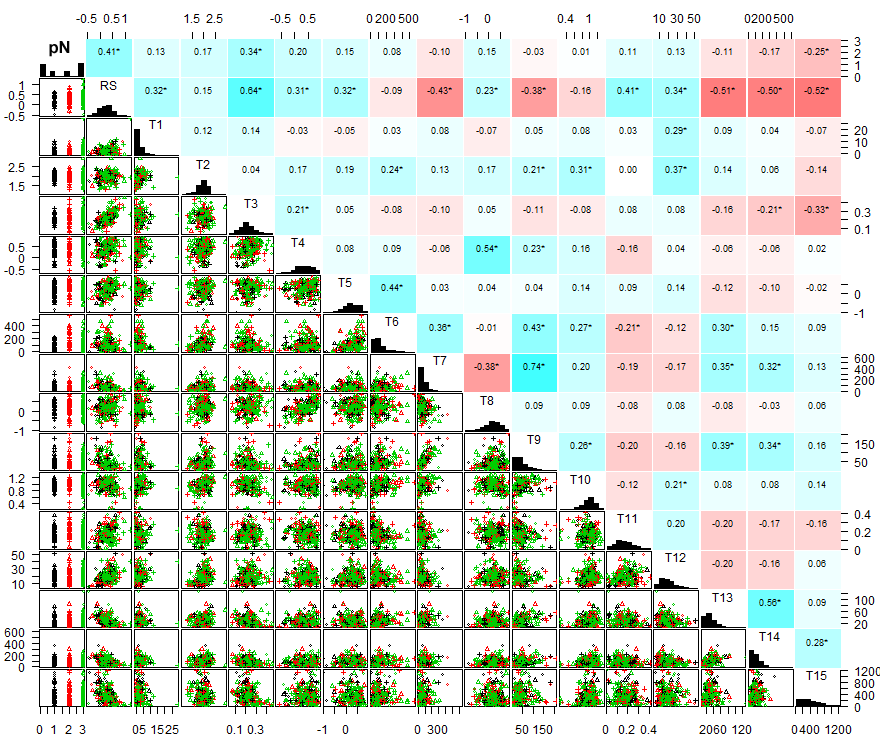


**Figure S5.** Scatterplot matrix of the interrelationship between the 15 radiomics features and Rad-score, pN stage in the internal validation cohort. The values denote Pearson correlation coefficients, with numbers closer to 1 identifying a better correlation. *p<0.0001. pN: pathological N stage; RS: Rad-score; T1 to T15 represent the 15 radiomics features.


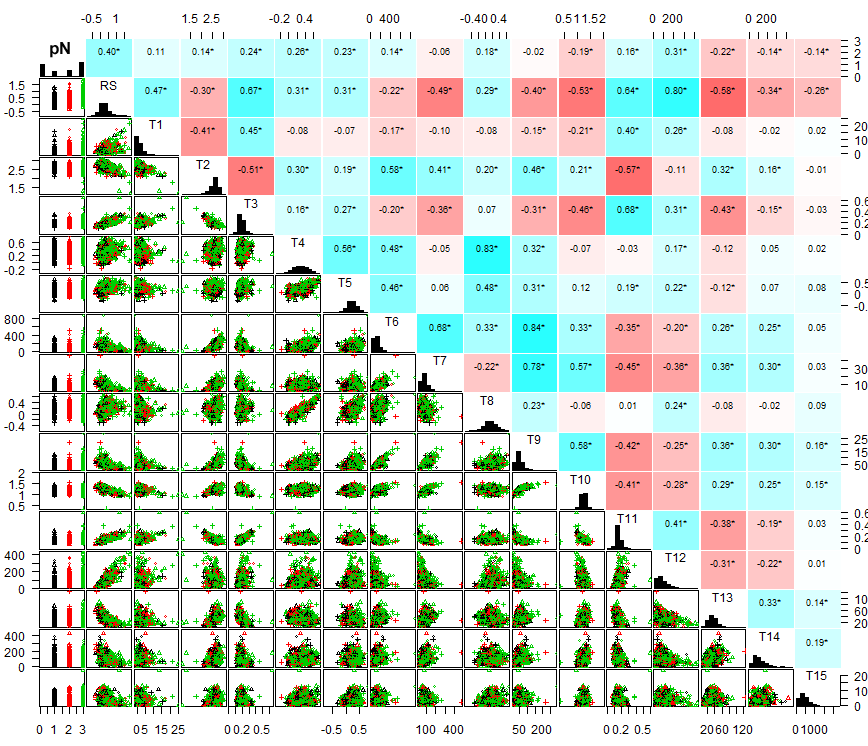


**Figure S6.** Scatterplot matrix of the interrelationship between the 15 radiomics features and Rad-score, pN stage in the external validation cohort. The values denote Pearson correlation coefficients, with numbers closer to 1 identifying a better correlation. *p<0.0001. pN: pathological N stage; RS: Rad-score; T1 to T15 represent the 15 radiomics features.


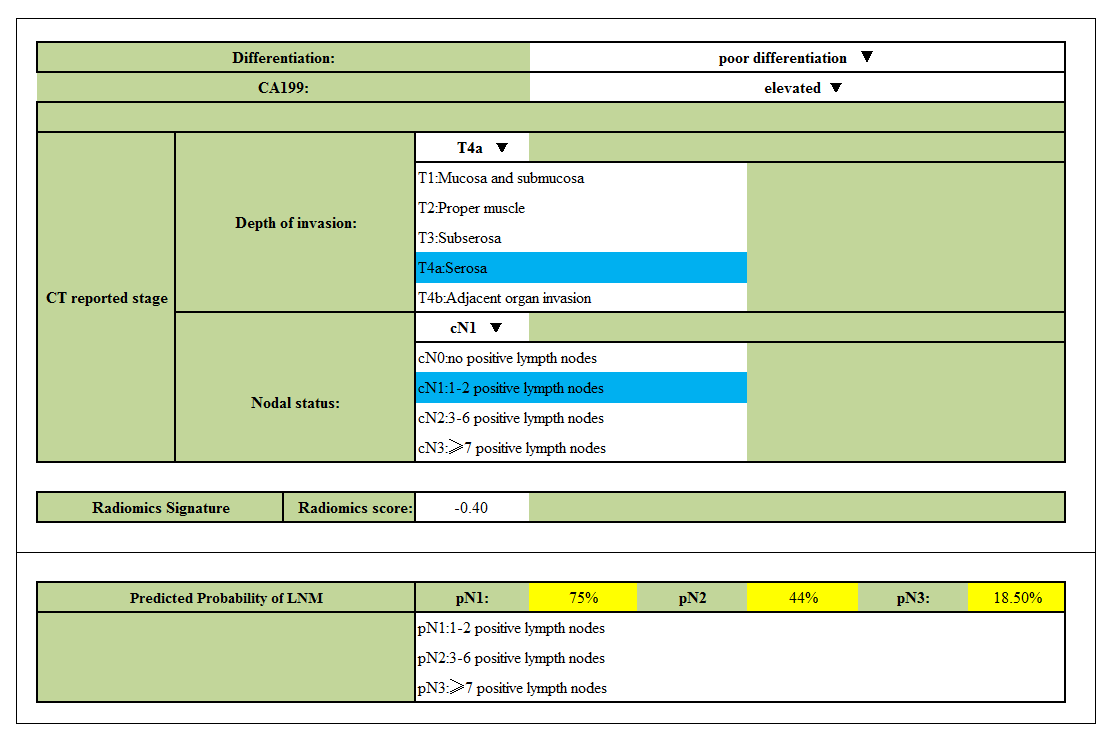


**Figure S7.** Calculating tool that can be used to help make clinical decisions regarding the probability of each pN stage for an individual patient. The system will calculate an individualized estimate of probability of each pN stage. (not online yet)


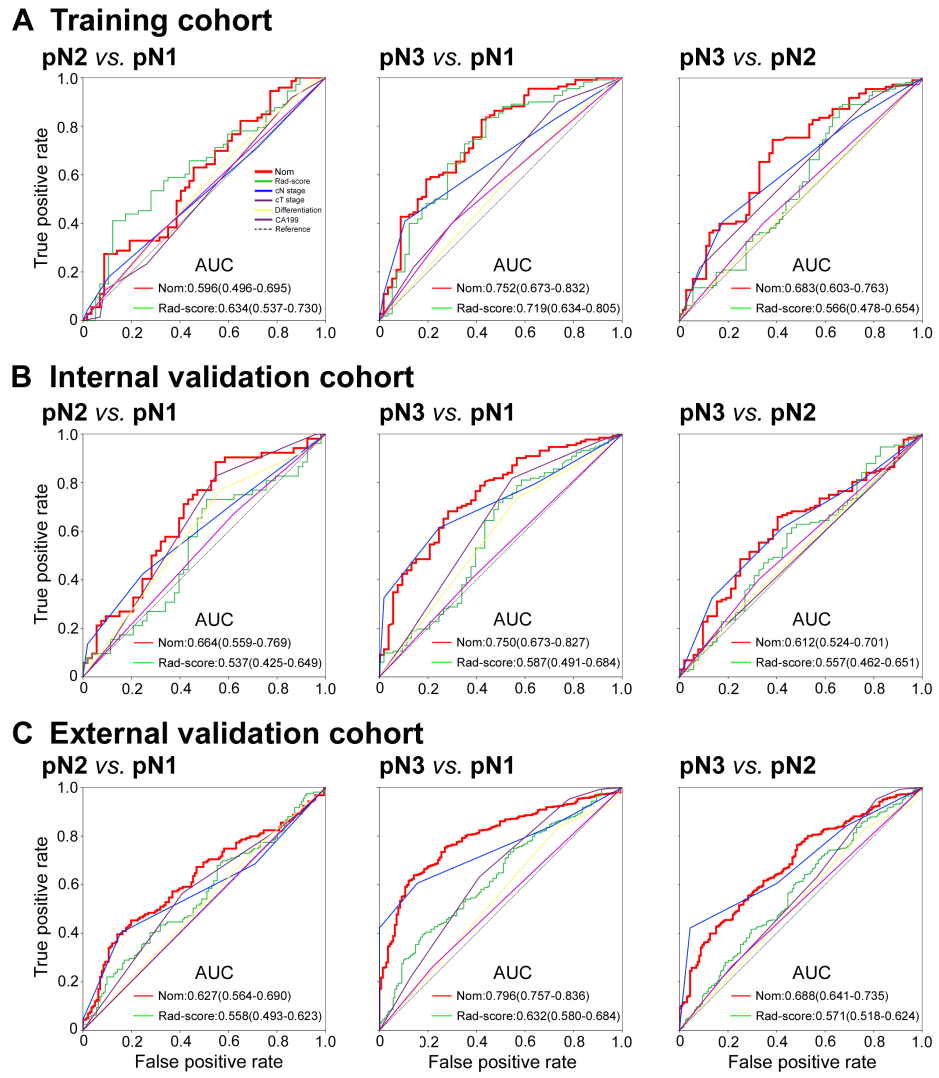


**Figure S8. Receiver operating characteristic (ROC) curves of pN2 vs pN1, pN3 vs pN1, and pN3 vs pN2 in each cohort**. pN2 *vs.* pN1: left panels; pN3 *vs.* pN1: middle panels; pN3 *vs.* pN2: right panels.


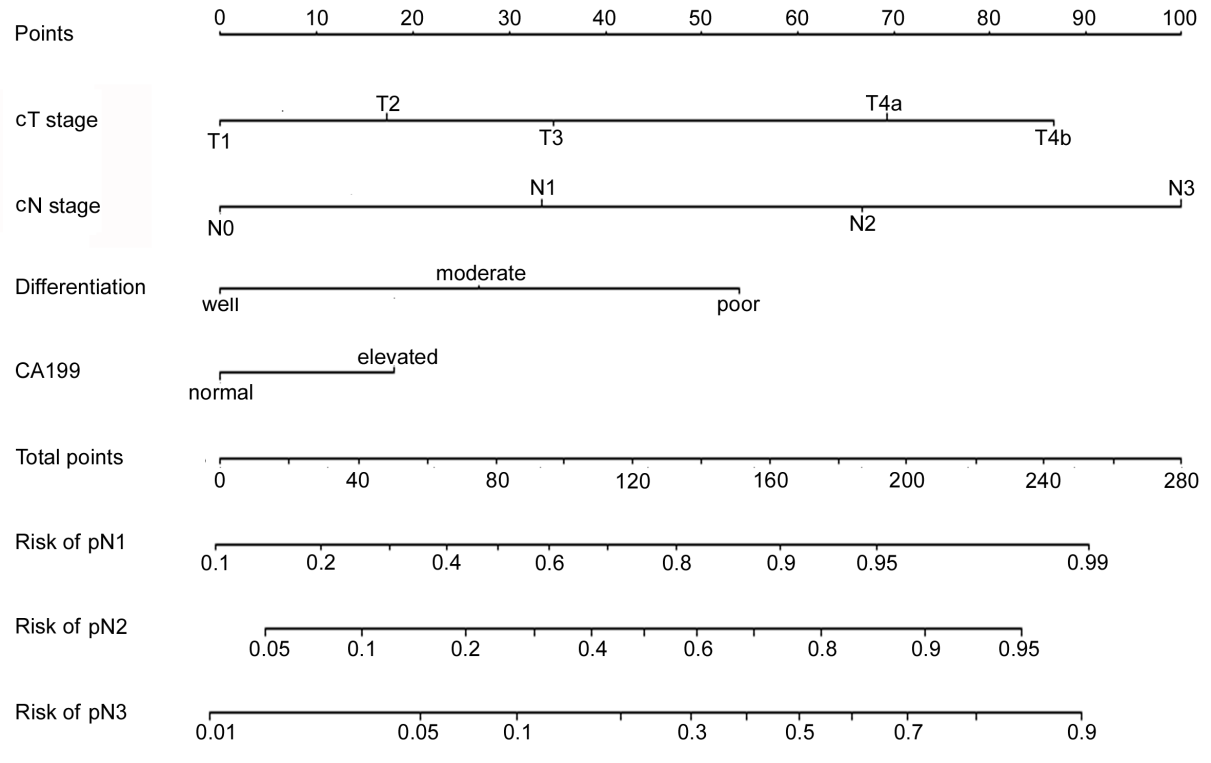


**Figure S9. Developed clinicopathological nomogram.** The clinicopathological nomogram was developed in the training cohort, with the cT stage and cN stage, differentiation status and CA199 level incorporated.

**Table S1**. Characteristics of patients in the training cohort.

| **Variables** | **Training Cohort (N=312)** | | | | | ***P*** |
| --- | --- | --- | --- | --- | --- | --- |
|  | Number | pN0(72) | pN1(57) | pN2(73) | pN3(110) |  |
| **Age (years)** |  |  | |  |  | 0.732 |
| ≥60 | 119 | 24(33.3) | 22(38.6) | 31(42.5) | 42(38.2) |  |
| < 60 | 193 | 48(66.7) | 35(61.4) | 42(57.5) | 68(61.8) |  |
| **Gender** |  |  |  |  |  | 0.921 |
| Male | 216 | 51(70.8) | 41(71.9) | 49(67.1) | 75(68.2) |  |
| Female | 96 | 21(29.2) | 16(28.1) | 24(32.9) | 35(31.8) |  |
| **Size** |  |  |  |  |  | 0.012 |
| ≥4cm | 168 | 28(38.9) | 34(59.6) | 37(50.7) | 69(62.7) |  |
| < 4cm | 144 | 44(61.1) | 23(40.4) | 36(49.3) | 41(37.3) |  |
| **Differentiation** |  |  |  |  |  | <0.0001 |
| Well | 28 | 21(29.2) | 3(5.3) | 1(1.4) | 3(2.7) |  |
| Moderate | 93 | 19(26.4) | 19(33.3) | 22(30.1) | 33(30.0) |  |
| Poor or undifferentiated | 191 | 32(44.4) | 35(61.4) | 50(68.5) | 74(67.3) |  |
| **Location** |  |  |  |  |  | 0.428 |
| Cardia | 64 | 11(15.3) | 12(21.1) | 17(23.3) | 24(21.8) |  |
| Body | 51 | 13(18.1) | 14(24.6) | 8(11.0) | 16(14.5) |  |
| Antrum | 154 | 40(55.6) | 22(38.6) | 35(47.9) | 57(51.8) |  |
| Whole | 43 | 8(11.1) | 9(15.8) | 13(17.8) | 13(11.8) |  |
| **CEA** |  |  |  |  |  | 0.292 |
| elevated | 59 | 11(15.3) | 7(12.3) | 16(21.9) | 25(22.7) |  |
| normal | 253 | 61(55.6) | 50(38.6) | 57(47.9) | 85(51.8) |  |
| **CA199** |  |  |  |  |  | 0.0002 |
| elevated | 93 | 7(9.7) | 17(29.8) | 25(34.2) | 44(40.0) |  |
| normal | 219 | 65(90.3) | 40(70.2) | 48(65.8) | 66(60.0) |  |
| **cT stage** |  |  |  |  |  | <0.0001 |
| T1 | 14 | 6(8.3) | 4(7.0) | 1(1.4) | 3(2.7) |  |
| T2 | 28 | 18(25.0) | 1(1.8) | 8(11.0) | 1(0.9) |  |
| T3 | 39 | 14(19.4) | 10(17.5) | 8(11.0) | 7(6.4) |  |
| T4a | 191 | 32(44.4) | 34(59.6) | 50(68.5) | 75(68.2) |  |
| T4b | 40 | 2(2.8) | 8(14.0) | 6(8.2) | 24(21.8) |  |
| **cN stage** |  |  |  |  |  | <0.0001 |
| N0 | 97 | 41(56.9) | 16(28.0) | 21(28.8) | 19(17.3) |  |
| N1 | 149 | 29(40.3) | 35(61.4) | 39(53.4) | 46(41.8) |  |
| N2 | 50 | 2(2.8) | 5(8.8) | 10(13.7) | 33(30.0) |  |
| N3 | 16 | 0(0) | 1(1.8) | 3(4.1) | 12(10.9) |  |
| **pT stage** |  |  |  |  |  | <0.0001 |
| pT1 | 20 | 14(19.5) | 4(7.0) | 2(2.7) | 0(0) |  |
| pT2 | 22 | 10(13.9) | 2(3.5) | 5(6.8) | 5(4.6) |  |
| pT3 | 18 | 8(11.1) | 4(7.0) | 2(2.7) | 4(3.6) |  |
| pT4a | 176 | 32(44.4) | 32(56.1) | 47(64.4) | 65(59.1) |  |
| pT4b | 76 | 8(11.1) | 15(26.4) | 17(23.3) | 36(32.7) |  |
| **Rad-score, mean(95%CI)** | 312 | -0.092(-0.170, -0.014) | 0.110(0.029, 0.191) | 0.234(0.174, 0.294) | 0.307(0.259, 0.354) | <0.0001 |

| Table S2. Characteristics of patients in the internal validation cohorts | | | | | | |
| --- | --- | --- | --- | --- | --- | --- |
| **Variables** | **Internal Validation Cohort (N=360)** | | | | | ***P*** |
|  | Number | pN0(123) | pN1(53) | pN2(52) | pN3(132) |  |
| **Age (years)** |  |  | |  |  | 0.96 |
| ≥60 | 154 | 53(43.1) | 21(39.6) | 22(42.3) | 58(43.9) |  |
| < 60 | 206 | 70(56.9) | 32(60.4) | 30(57.7) | 74(56.1) |  |
| **Gender** |  |  |  |  |  | 0.836 |
| Male | 256 | 89(72.4) | 35(66.0) | 38(73.1) | 94(71.2) |  |
| Female | 104 | 34(27.6) | 18(34.0) | 14(26.9) | 38(28.8) |  |
| **Size** |  |  |  |  |  | 0.221 |
| ≥4cm | 163 | 57(48.7) | 28(53.8) | 17(34.7) | 61(50.0) |  |
| < 4cm | 177 | 60(51.3) | 24(46.2) | 32(65.3) | 61(50.0) |  |
| **Differentiation** |  |  |  |  |  | <0.0001 |
| Well | 40 | 27(29.2) | 3(5.3) | 3(1.4) | 7(2.7) |  |
| Moderate | 100 | 43(35.0) | 20(37.7) | 9(17.3) | 28(21.2) |  |
| Poor or  undifferentiated | 220 | 53(43.1) | 30(56.6) | 40(76.9) | 97(73.5) |  |
| **Location** |  |  |  |  |  | 0.287 |
| Cardia | 81 | 39(24.4) | 11(20.8) | 9(17.3) | 31(23.5) |  |
| Body | 93 | 23(18.7) | 19(35.8) | 14(26.9) | 37(28.0) |  |
| Antrum | 168 | 65(52.8) | 22(41.5) | 24(46.2) | 57(43.2) |  |
| Whole | 18 | 5(4.1) | 1(1.9) | 5(9.6) | 7(5.3) |  |
| **CEA** |  |  |  |  |  | 0.542 |
| elevated | 112 | 42(34.1) | 17(32.1) | 18(34.6) | 35(26.5) |  |
| normal | 248 | 81(65.9) | 36(67.9) | 34(65.4) | 97(73.5) |  |
| **CA199** |  |  |  |  |  | 0.0003 |
| elevated | 110 | 20(16.3) | 20(37.7) | 17(32.7) | 53(40.2) |  |
| normal | 250 | 103(83.7) | 33(62.3) | 35(67.3) | 79(59.8) |  |
| **cT stage** |  |  |  |  |  | <0.0001 |
| T1 | 30 | 27(22.0) | 2(3.8) | 0(0.0) | 1(0.8) |  |
| T2 | 44 | 32(26.0) | 5(9.4) | 2(3.8) | 5(3.8) |  |
| T3 | 68 | 26(21.1) | 17(32.1) | 7(13.5) | 18(13.6) |  |
| T4a | 183 | 37(30.1) | 23(43.4) | 36(69.2) | 87(65.9) |  |
| T4b | 35 | 1(0.8) | 6(11.3) | 7(13.5) | 21(15.9) |  |
| **cN stage** |  |  |  |  |  | <0.0001 |
| N0 | 146 | 86(69.9) | 19(35.8) | 14(26.9) | 27(20.5) |  |
| N1 | 75 | 14(11.4) | 21(39.6) | 16(30.8) | 24(18.2) |  |
| N2 | 85 | 20(16.3) | 12(22.6) | 15(28.8) | 38(28.8) |  |
| N3 | 54 | 3(2.4) | 1(1.9) | 7(13.5) | 43(32.6) |  |
| **pT stage** |  |  |  |  |  | <0.0001 |
| pT1 | 48 | 44(35.7) | 3(5.7) | 0(0) | 1(0.8) |  |
| pT2 | 29 | 17(13.8) | 5(9.4) | 2(3.8) | 5(3.8) |  |
| pT3 | 55 | 23(18.7) | 7(13.2) | 8(15.4) | 17(12.9) |  |
| pT4a | 165 | 34(27.7) | 31(58.5) | 30(57.7) | 70(53.0) |  |
| pT4b | 63 | 5(4.1) | 7(13.2) | 12(23.1) | 39(29.5) |  |
| **Rad-score, mean (95%CI)** | 360 | 0.075(0.037, 0.114) | 0.219(0.151, 0.288) | 0.258(0.185, 0.331) | 0.305(0.264, 0.347) | <0.0001 |

| **Table S3**. Characteristics of patients in the external validation cohort. | | | | | | |
| --- | --- | --- | --- | --- | --- | --- |
| **Variables** | **External Validation Cohort (N=1,017)** | | | | | ***P*** |
|  | Number | pN0(321) | pN1(141) | pN2(159) | pN3(396) |  |
| **Age (years)** |  |  | |  |  | 0.377 |
| ≥60 | 405 | 122(38.0) | 62(44.0) | 70(44.0) | 151(38.1) |  |
| < 60 | 612 | 199(62.0) | 79(56.0) | 89(56.0) | 245(61.9) |  |
| **Gender** |  |  |  |  |  | 0.431 |
| Male | 681 | 223(69.5) | 98(69.5) | 106(66.7) | 254(64.1) |  |
| Female | 336 | 98(30.5) | 43(30.5) | 53(33.3) | 142(35.9) |  |
| **Size** |  |  |  |  |  | <0.0001 |
| ≥4cm | 634 | 138(43.0) | 77(54.6) | 111(69.8) | 308(77.8) |  |
| < 4cm | 383 | 183(57.0) | 64(45.4) | 48(30.2) | 88(22.2) |  |
| **Differentiation** |  |  |  |  |  | <0.0001 |
| Well | 121 | 80(25.0) | 7(5.0) | 7(4.4) | 27(6.8) |  |
| Moderate | 181 | 85(26.5) | 30(21.3) | 29(18.2) | 37(9.3) |  |
| Poor or undifferentiation | 715 | 159(49.5) | 104(73.7) | 123(77.4) | 332(83.9) |  |
| **Location** |  |  |  |  |  | 0.0002 |
| Cardia | 276 | 86(26.8) | 42(29.8) | 52(32.7) | 96(24.2) |  |
| Body | 230 | 64(19.9) | 30(21.3) | 36(22.6) | 100(25.3) |  |
| Antrum | 452 | 162(50.5) | 62(44.0) | 68(42.8) | 160(40.4) |  |
| Whole | 59 | 9(2.8) | 7(4.9) | 3(1.9) | 40(10.1) |  |
| **CEA** |  |  |  |  |  | <0.0001 |
| elevated | 191 | 32(10.0) | 17(12.1) | 39(24.5) | 103(26.0) |  |
| nomal | 826 | 289(90.0) | 124(87.9) | 120(75.5) | 293(74.0) |  |
| **CA199** |  |  |  |  |  | <0.0001 |
| elevated | 184 | 15(4.7) | 31(22.0) | 34(21.4) | 104(26.2) |  |
| nomal | 833 | 306(95.3) | 110(78.0) | 125(78.6) | 292(73.8) |  |
| **cT stage** | |  |  |  |  | <0.0001 |
| T1 | 129 | 98(30.5) | 13(9.2) | 15(9.5) | 3(0.8) |  |
| T2 | 118 | 69(21.5) | 18(12.8) | 15(9.5) | 16(4.0) |  |
| T3 | 291 | 73(22.7) | 52(36.9) | 39(24.5) | 127(32.1) |  |
| T4a | 324 | 58(18.1) | 40(28.4) | 62(39.0) | 164(41.4) |  |
| T4b | 155 | 23(7.2) | 18(12.7) | 28(17.5) | 86(21.7) |  |
| **cN stage** | |  |  |  |  | <0.0001 |
| N0 | 379 | 225(70.1) | 41(29.1) | 50(31.4) | 63(15.9) |  |
| N1 | 268 | 52(16.2) | 78(55.3) | 45(28.3) | 93(23.5) |  |
| N2 | 178 | 26(8.1) | 22(15.6) | 57(35.9) | 73(18.4) |  |
| N3 | 192 | 18(5.6) | 0(0.0) | 7(4.4) | 167(42.2) |  |
| **pT stage** |  |  |  |  |  | <0.0001 |
| pT1 | 123 | 96(29.9) | 15(10.7) | 10(6.3) | 2(0.5) |  |
| pT2 | 118 | 61(19.0) | 21(14.9) | 20(12.6) | 16(4.0) |  |
| pT3 | 229 | 54(16.8) | 47(33.3) | 49(30.8) | 79(20.0) |  |
| pT4a | 463 | 102(31.8) | 47(33.3) | 66(41.5) | 248(62.6) |  |
| pT4b | 84 | 8(2.5) | 11(7.8) | 14(8.8) | 51(12.9) |  |
| **Rad-score, mean (95%CI)** | 1,017 | 0.080(0.053, 0.106) | 0.248(0.202, 0.294) | 0.317(0.266, 0.369) | 0.392(0.358, 0.425) | <0.0001 |

| **Table S4**. Stratified analysis of the association between the radiomics signature and N stage in the training cohort | | | | | | |
| --- | --- | --- | --- | --- | --- | --- |
| **Variables** | **Rad-score (Training Cohort, N=312)** | | | | | ***P*** |
|  | Number | pN0(72) | pN1(57) | pN2(73) | pN3(110) |  |
| **Age (years)** |  |  | |  |  |  |
| ≥60 | 119 | -0.139(-0.257, 0.021) | 0.180(0.024, 0.336) | 0.166(0.059, 0.273) | 0.285(0.206, 0.364) | <0.0001 |
| < 60 | 193 | -0.069(-0.172, 0.035) | 0.065(-0.027, 0.158) | 0.284(0.214, 0.354) | 0.320(0.259, 0.381) | <0.0001 |
| **Gender** |  |  |  |  |  |  |
| Male | 216 | -0.056(-0.147, 0.034) | 0.133(0.036, 0.230) | 0.201(0.122, 0.279) | 0.319(0.271, 0.366) | <0.0001 |
| Female | 96 | -0.179(-0.341, -0.016) | 0.049(-0.112, 0.211) | 0.302(0.212, 0.393) | 0.281(0.168, 0.394) | <0.0001 |
| **Size** |  |  |  |  |  |  |
| ≥4cm | 168 | -0.063(-0.192, 0.066) | 0.169(0.049, 0.290) | 0.283(0.188, 0.378) | 0.336(0.275, 0.397) | <0.0001 |
| < 4cm | 144 | -0.110(-0.213, 0.008) | 0.022(-0.069, 0.113) | 0.184(0.109, 0.259) | 0.257(0.180, 0.333) | <0.0001 |
| **Differentiation** |  |  |  |  |  |  |
| Well | 28 | -0.235(-0.403, 0.066) | 0.002(-0.027, 0.301) | 0.303 | 0.271(0.025, 0.518) | <0.0001 |
| Moderate | 93 | -0.042(-0.212, 0.128) | 0.147(-0.025, 0.319) | 0.254(0.160, 0.348) | 0.322(0.238, 0.406) | <0.0001 |
| Poor or  undifferentiated | 191 | -0.028(-0.126, 0.070) | 0.099(-0.001, 0.199) | 0.224(0.144, 0.304) | 0.301(0.240, 0.363) | <0.0001 |
| **Location** |  |  |  |  |  |  |
| Cardia | 64 | -0.141(-0.311, 0.028) | 0.133(0.000, 0.267) | 0.189(0.083, 0.294) | 0.265(0.176, 0.354) | <0.0001 |
| Body | 51 | -0.354(-0.589, 0.119) | 0.000(-0.162, 0.162) | 0.275(0.102, 0.448) | 0.310(0.194, 0.426) | <0.0001 |
| Antrum | 154 | -0.046(-0.142, 0.501) | 0.128(-0.023, 0.279) | 0.221(0.115, 0.326) | 0.323(0.245, 0.401) | <0.0001 |
| Whole | 43 | 0.172(-0.015, 0.359) | 0.205(-0.069, 0.479) | 0.304(0.174, 0.434) | 0.301(0.240, 0.363) | 0.48 |
| **CEA** |  |  |  |  |  |  |
| elevated | 59 | 0.163(-0.035, 0.360) | 0.168(-0.046, 0.382) | 0.276(0.128, 0.427) | 0.231(0.110, 0.351) | 0.713 |
| normal | 253 | -0.138(-0.220, 0.056) | 0.102(0.012, 0.191) | 0.222(0.154, 0.289) | 0.329(0.278, 0.380) | <0.0001 |
| **CA199** |  |  |  |  |  |  |
| elevated | 93 | -0.237(-0.698, 0.224) | 0.115(-0.033, 0.262) | 0.257(0.146, 0.368) | 0.273(0.178, 0.368) | 0.001 |
| normal | 219 | -0.076(-0.154, 0.001) | 0.108(0.006, 0.209) | 0.222(0.148, 0.296) | 0.329(0.279, 0.379) | <0.0001 |
| **cT stage** |  |  |  |  |  |  |
| T1+T2 | 42 | -0.010(-0.140, 0.120) | 0.034(-0.199, 0.268) | 0.307(0.139, 0.474) | 0.325(0.130, 0.521) | 0.012 |
| T3 | 39 | -0.252(-0.430, 0.074 | 0.098(-0.305, 0.108) | 0.307(0.139, 0.476) | 0.173(-0.269, 0.615) | 0.002 |
| T4a | 191 | -0.090(-0.218, 0.039) | 0.178(0.069, 0.287) | 0.193(0.113, 0.273) | 0.333(0.275, 0.392) | <0.0001 |
| T4b | 40 | 0.005(-0.817, 0.827) | 0.126(-0.094, 0.347) | 0.370(0.295, 0.446) | 0.259(0.197, 0.321) | 0.018 |
| **cN stage** |  |  |  |  |  |  |
| N0 | 97 | -0.031(-0.111, 0.048) | 0.174(0.041, 0.307) | 0.120(0.002, 0.239) | 0.319(0.215, 0.423) | <0.0001 |
| N1 | 149 | -0.163(-0.321, 0.005) | 0.071(-0.043, 0.186) | 0.276(0.192, 0.360) | 0.347(0.271, 0.423) | <0.0001 |
| N2 | 50 | -0.310(-0.505, 0.094) | 0.081(-0.187, 0.350) | 0.311(0.149, 0.474) | 0.297(0.235, 0.360) | 0.0003 |
| N3 | 16 | 0.573 | 0.229(-0.267, 0.724) | 0.159(-0.098, 0.416) | 0.198(0.001, 0.394) | 0.584 |
| **pT Stage** |  |  |  |  |  |  |
| pT1 | 20 | -0.293(-0.465, -0.122) | 0.031(-0.145, 0.206) | 0.441 | 0 | 0.004 |
| pT2 | 22 | 0.055(-0.211, 0.322) | -0.030 | 0.024(-4.150, 4.198) | 0.317(0.010, 0.626) | 0.507 |
| pT3 | 18 | -0.137(-0.401, 0.127) | -0.078(-0.630, 0.474) | 0.271(-2.212, 2.753) | 0.007(-0.681, 0.696) | 0.526 |
| pT4a | 176 | -0.076(-0.195, 0.044) | 0.170(0.052, 0.287) | 0.181(0.104, 0.257) | 0.325(0.270, 0.381) | <0.0001 |
| pT4b | 76 | 0.054(-0.141, 0.250) | 0.072(-0.090, 0.233) | 0.338(0.218, 0.459) | 0.329(0.239, 0.419) | 0.002 |

| **Table S5**. Stratified analysis of the association between the radiomics signature and LN metastasis in the  internal validation cohort. | | | | | | |
| --- | --- | --- | --- | --- | --- | --- |
| **Variables** | **Rad-score (Internal Validation Cohort, N=360)** | | | | | ***P*** |
|  | Number | pN0(123) | pN1(53) | pN2(52) | pN3(132) |  |
| **Age (years)** |  |  | |  |  |  |
| ≥60 | 154 | 0.102(0.040, 0.164) | 0.273(0.162, 0.385) | 0.326(0.212, 0.439) | 0.342(0.288, 0.395) | <0.0001 |
| < 60 | 206 | 0.055(0.005, 0.105) | 0.184(0.095, 0.273) | 0.208(0.113, 0.304) | 0.277(0.215, 0.339) | <0.0001 |
| **Gender** |  |  |  |  |  |  |
| Male | 256 | 0.101(0.059, 0.142) | 0.214(0.126, 0.302) | 0.261(0.183, 0.340) | 0.322(0.273, 0.370) | <0.0001 |
| Female | 104 | 0.009(-0.080, 0.097) | 0.229(0.111, 0.347) | 0.249(0.062, 0.435) | 0.265(0.184, 0.347) | 0.0003 |
| **Size** |  |  |  |  |  |  |
| ≥4cm | 163 | 0.099(0.046, 0.152) | 0.208(0.120, 0.296) | 0.290(0.143, 0.437) | 0.265(0.193, 0.338) | 0.002 |
| < 4cm | 177 | 0.060(-0.001, 0.120) | 0.225(0.108, 0.342) | 0.210(0.126, 0.295) | 0.338(0.285, 0.390) | <0.0001 |
| **Differentiation** |  |  |  |  |  |  |
| Well | 40 | 0.037(-0.053, 0.127) | 0.187(-0.756, 1.129) | 0.205(-0.372, 0.782) | 0.246(0.037, 0.455) | 0.162 |
| Moderate | 100 | 0.055(-0.005, 0.115) | 0.258(0.139, 0.377) | 0.225(0.041, 0.409) | 0.289(0.190, 0.389) | 0.0002 |
| Poor or  undifferentiated | 220 | 0.111(0.049, 0.173) | 0.197(0.109, 0.286) | 0.269(0.183, 0.356) | 0.314(0.266, 0.363) | <0.0001 |
| **Location** |  |  |  |  |  |  |
| Cardia | 81 | 0.087(0.015, 0.160) | 0.213(-0.008, 0.433) | 0.266(0.168, 0.364) | 0.294(0.229, 0.358) | 0.002 |
| Body | 93 | 0.076(0.000, 0.152) | 0.251(0.138, 0.365) | 0.321(0.190, 0.453) | 0.305(0.214, 0.397) | 0.003 |
| Antrum | 168 | 0.074(0.014, 0.134) | 0.201(0.100, 0.301) | 0.192(0.061, 0.322) | 0.304(0.032, 0.241) | <0.0001 |
| Whole | 18 | 0.013(-0.255, 0.280) | 0.103 | 0.386(0.093, 0.679) | 0.369(0.023, 0.713) | 0.186 |
| **CEA** |  |  |  |  |  |  |
| elevated | 112 | 0.100(0.042, 0.157) | 0.221(0.096, 0.345) | 0.238(0.120, 0.357) | 0.223(0.162, 0.285) | 0.018 |
| normal | 248 | 0.062(0.012, 0.114) | 0.219(0.133, 0.305) | 0.269(0.173, 0.364) | 0.335(0.283, 0.386) | <0.0001 |
| **CA199** |  |  |  |  |  |  |
| elevated | 110 | -0.237(-0.698, 0.224) | 0.115(-0.033, 0.262) | 0.257(0.146, 0.368) | 0.273(0.178, 0.368) | 0.001 |
| normal | 250 | 0.074(0.031, 0.117) | 0.201(0.109, 0.293) | 0.259(0.169, 0.350) | 0.339(0.290, 0.389) | <0.0001 |
| **cT stage** |  |  |  |  |  |  |
| T1+T2 | 30 | 0.089(0.029, 0.150) | 0.323(0.117, 0.529) | 0.024(-0.304, 0.221) | 0.357(0.141, 0.572) | 0.009 |
| T3 | 68 | 0.078(0.014, 0.141) | 0.111(-0.038, 0.260) | 0.230(0.046, 0.415) | 0.240(0.085, 0.395) | 0.132 |
| T4a | 183 | 0.051(-0.026, 0.128) | 0.218(0.137, 0.299) | 0.270(0.185, 0.355) | 0.292(0.247, 0.336) | <0.0001 |
| T4b | 35 | 0.078 | 0.410(0.156, 0.664) | 0.290(-0.017, 0.596) | 0.404(0.272, 0.536) | 0.594 |
| **cN stage** |  |  |  |  |  |  |
| N0 | 146 | 0.080(0.032, 0.128) | 0.149(0.021, 0.276) | 0.293(0.139, 0.447) | 0.325(0.243, 0.406) | <0.0001 |
| N1 | 75 | 0.080(-0.054, 0.214) | 0.257(0.149, 0.364) | 0.189(0.051, 0.328) | 0.267(0.147, 0.387) | 0.146 |
| N2 | 85 | 0.081(0.021, 0.142) | 0.245(0.094, 0.397) | 0.234(0.074, 0.395) | 0.373(0.293, 0.453) | 0.0003 |
| N3 | 54 | ﹣0.117(-0.378, 0.290) | 0.468 | 0.395(0.267, 0.524) | 0.255(0.185, 0.325) | 0.014 |
| **pT Stage** |  |  |  |  |  |  |
| pT1 | 48 | 0.070(0.013, 0.128) | 0.418(0.006, 0.830) | / | 0.281 | 0.009 |
| pT2 | 29 | 0.061(-0.050, 0.172) | 0.104(-0.338, 0.546) | 0.024(-4.150, 4.198) | 0.317(0.010, 0.626) | 0.291 |
| pT3 | 55 | 0.124(0.041, 0.206) | 0.097(-0.135, 0.329) | 0.228(0.044, 0.412) | 0.331(0.211, 0.451) | 0.019 |
| pT4a | 165 | 0.060(-0.033, 0.152) | 0.219(0.139, 0.298) | 0.271(0.184, 0.357) | 0.294(0.240, 0.348) | <0.0001 |
| pT4b | 63 | 0.048(-0.236, 0.332) | 0.343(0.092, 0.594) | 0.285(0.073, 0.497) | 0.314(0.223, 0.404) | 0.262 |

| **Table S6**. Stratified analysis of the association between the radiomics signature and N stage in the external  validation cohort. | | | | | | |
| --- | --- | --- | --- | --- | --- | --- |
| **Variables** | **Rad-score (External Validation Cohort, N=1,017)** | | | | | ***P*** |
|  | Number | pN0(321) | pN1(141) | pN2(159) | pN3(396) |  |
| **Age (years)** |  |  | |  |  |  |
| ≥60 | 405 | 0.093(0.053, 0.133) | 0.231(0.152, 0.310) | 0.357(0.270, 0.444) | 0.418(0.357, 0.478) | <0.0001 |
| < 60 | 612 | 0.072(0.037, 0.107) | 0.262(0.207, 0.316) | 0.286(0.224, 0.348) | 0.375(0.335, 0.415) | <0.0001 |
| **Gender** |  |  |  |  |  |  |
| Male | 681 | 0.091(0.059, 0.123) | 0.243(0.185, 0.301) | 0.353(0.284, 0.421) | 0.414(0.371, 0.457) | <0.0001 |
| Female | 336 | 0.055(0.07, 0.103) | 0.259(0.185, 0.334) | 0.246(0.176, 0.315) | 0.351(0.297, 0.406) | <0.0001 |
| **Size** |  |  |  |  |  |  |
| ≥4cm | 634 | 0.098(0.057, 0.140) | 0.261(0.202, 0.320) | 0.345(0.280, 0.410) | 0.417(0.379, 0.455) | 0.002 |
| < 4cm | 383 | 0.066(0.031, 0.101) | 0.232(0.160, 0.305) | 0.252(0.171, 0.334) | 0.302(0.231, 0.372) | <0.0001 |
| **Differentiation** |  |  |  |  |  |  |
| Well | 121 | 0.126(0.066, 0.187) | 0.308(-0.067, 0.683) | 0.360(-0.066, 0.785) | 0.288(0.135, 0.440) | 0.04 |
| Moderate | 181 | 0.138(0.092, 0.183) | 0.250(0.172, 0.328) | 0.354(0.206, 0.502) | 0.341(0.232, 0.451) | <0.0001 |
| Poor or  undifferentiated | 715 | 0.025(-0.012, 0.061) | 0.243(0.188, 0.298) | 0.306(0.251, 0.361) | 0.406(0.369, 0.442) | <0.0001 |
| **Location** |  |  |  |  |  |  |
| Cardia | 276 | 0.080(0.030, 0.130) | 0.234(0.126, 0.342) | 0.359(0.241, 0.476) | 0.411(0.331, 0.491) | <0.0001 |
| Body | 230 | 0.025(-0.031, 0.081) | 0.315(0.216, 0.413) | 0.240(0.160, 0.320) | 0.370(0.308, 0.431) | <0.0001 |
| Antrum | 452 | 0.093(0.057, 0.130) | 0.216(0.162, 0.269) | 0.313(0.245, 0.382) | 0.382(0.333, 0.430) | <0.0001 |
| Whole | 59 | 0.227(-0.095, 0.549) | 0.338(0.039, 0.636) | 0.602(0.330, 0.875) | 0.439(0.316, 0.562) | 0.347 |
| **CEA** |  |  |  |  |  |  |
| elevated | 191 | 0.074(-0.005, 0.154) | 0.238(0.063, 0.412) | 0.361(0.254, 0.467) | 0.378(0.317, 0.440) | <0.0001 |
| normal | 826 | 0.080(0.052, 0.109) | 0.249(0.202, 0.297) | 0.302(0.244, 0.362) | 0.396(0.356, 0.436) | <0.0001 |
| **CA199** |  |  |  |  |  |  |
| elevated | 184 | -0.237(-0.698, 0.224) | 0.115(-0.033, 0.262) | 0.257(0.146, 0.368) | 0.273(0.178, 0.368) | 0.001 |
| normal | 833 | 0.074(0.031, 0.117) | 0.201(0.109, 0.293) | 0.259(0.169, 0.350) | 0.339(0.290, 0.389) | <0.0001 |
| **cT stage** |  |  |  |  |  |  |
| T1 | 129 | 0.077(0.308, 0.123) | 0.210(0.124, 0.296) | 0.278(0.112, 0.443) | 0.189(-0.032, 0.411) | 0.007 |
| T2 | 118 | 0.100(0.031, 0.169) | 0.197(0.070, 0.323) | 0.213(0.115, 0.311) | 0.241(0.098, 0.384) | 0.143 |
| T3 | 291 | 0.121(0.064, 0.177) | 0.241(0.170, 0.313) | 0.302(0.197, 0.407) | 0.419(0.349, 0.490) | <0.0001 |
| T4a | 324 | 0.018(-0.038, 0.074) | 0.249(0.148, 0.350) | 0.340(0.245, 0.435) | 0.397(0.347, 0.448) | <0.0001 |
| T4b | 155 | 0.058(-0.016, 0.132) | 0.344(0.188, 0.500) | 0.364(0.245, 0.484) | 0.375(0.316, 0.434) | <0.0001 |
| **cN stage** |  |  |  |  |  |  |
| N0 | 379 | 0.065(0.036, 0.094) | 0.290(0.215, 0.366) | 0.336(0.236, 0.436) | 0.366(0.289, 0.444) | <0.0001 |
| N1 | 268 | 0.156(0.095, 0.216) | 0.269(0.200, 0.337) | 0.347(0.235, 0.459) | 0.315(0.254, 0.376) | 0.006 |
| N2 | 178 | 0.073(-0.088, 0.235) | 0.095(0.023, 0.167) | 0.305(0.231, 0.378) | 0.421(0.333, 0.509) | <0.0001 |
| N3 | 192 | 0.056(-0.038, 0.149) | / | 0.086(-0.026, 0.198) | 0.431(0.377, 0.485) | <0.0001 |
| **pT Stage** |  |  |  |  |  |  |
| pT1 | 123 | 0.063(0.018, 0.108) | 0.158(0.072, 0.243) | 0.206(0.051, 0.361) | 0.259(-0.085, 0.604) | 0.067 |
| pT2 | 118 | 0.068(0.011, 0.124) | 0.184(0.072, 0.296) | 0.265(0.144, 0.386) | 0.245(0.105, 0.385) | 0.003 |
| pT3 | 229 | 0.095(0.033, 0.156) | 0.209(0.132, 0.286) | 0.217(0.139, 0.294) | 0.359(0.290, 0.429) | <0.0001 |
| pT4a | 463 | 0.082(0.030, 0.134) | 0.305(0.213, 0.398) | 0.408(0.313, 0.503) | 0.411(0.367, 0.457) | <0.0001 |
| pT4b | 84 | 0.253(-0.072, 0.578) | 0.415(0.262, 0.569) | 0.393(0.245, 0.542) | 0.394(0.310, 0.477) | 0.615 |

| **Table S7. Univariate association of Rad-score, clinicopathological characteristics with Lymph node metastasis in the training, internal**  **and external validation cohorts.** | | | | | | | | |
| --- | --- | --- | --- | --- | --- | --- | --- | --- |
| **Variables** | **pN1 vs. pN0** | |  | **pN2 vs. pN0** | |  | **pN3 vs. pN0** | |
|  | HR (95%CI) | *P* |  | HR (95%CI) | *P* |  | HR (95%CI) | *P* |
| **Training cohort** |  |  |  |  |  |  |  |  |
| **Rad-score** | **9.551 (2.712-33.64)** | **<0.0001** |  | **51.96 (13.58-198.8)** | **<0.0001** |  | **144.84 (38.00-552.1)** | **<0.0001** |
| Age(years) (≥60 vs. <60) | 1.257 (0.609-2.594) | 0.536 |  | 1.467 (0.752-2.899) | 0.258 |  | 1.206 (0.646-2.252) | 0.557 |
| Gender (male vs. female) | 1.055 (0.489-2.278) | 0.891 |  | 0.841 (0.415-1.701) | 0.629 |  | 0.882 (0.462-1.686) | 0.705 |
| Tumor size | 2.323 (1.142-4.726) | 0.020 |  | 1.615 (0.835-3.125) | 0.154 |  | 2.645 (1.435-4.874) | 0.020 |
| Differentiation | 2.313 (1.365-3.920) | 0.002 |  | 3.184 (1.868-5.424) | <0.0001 |  | 2.932 (1.841-4.670) | <0.0001 |
| CEA | 0.776 (0.280-2.150) | 0.626 |  | 1.557 (0.666-3.636) | 0.307 |  | 1.631 (0.746-3.564) | 0.22 |
| CA199 | 3.946 (1.505-10.351) | 0.005 |  | 4.836 (1.932-12.104) | 0.001 |  | 6.190 (2.599-14.746) | <0.0001 |
| cT stage | 1.518 (1.180-1.952) | 0.001 |  | 1.540 (1.217-1.948) | <0.0001 |  | 2.304 (1.756-3.022) | <0.0001 |
| cN stage | 2.449 (1.418-4.228) | 0.001 |  | 2.886 (1.717-4.850) | <0.0001 |  | 5.484 (3.288-9.146) | <0.0001 |
|  |  |  |  |  |  |  |  |  |
| **Internal validation cohort** |  |  |  |  |  |  |  |  |
| **Rad-score** | **14.537 (3.418-61.83)** | **<0.0001** |  | **29.436 (6.70-129.36)** | **<0.0001** |  | **68.17 (19.95-232.9)** | **<0.0001** |
| Age(years) (≥60 vs. <60) | 0.867 (0.450-1.670) | 0.669 |  | 0.969 (0.503-1.866) | 0.969 |  | 1.035 (0.631-1.699) | 0.891 |
| Gender (male vs. female) | 0.743 (0.372-1.484) | 0.400 |  | 1.037 (0.500-2.150) | 0.922 |  | 0.945 (0.547-1.631) | 0.839 |
| Tumor size | 1.228 (0.638-2.364) | 0.539 |  | 0.599 (0.280-1.116) | 0.099 |  | 1.053 (0.634-1.748) | 0.843 |
| Differentiation | 1.759 (1.104-2.804) | 0.018 |  | 3.062 (1.737-5.400) | <0.0001 |  | 2.773 (1.871-4.110) | <0.0001 |
| CEA | 0.911 (0.458-1.810) | 0.790 |  | 1.021 (0.516-2.020) | 0.952 |  | 0.696 (0.407-1.190) | 0.186 |
| CA199 | 3.121 (1.499-6.499) | 0.002 |  | 2.501 (1.180-5.305) | 0.017 |  | 3.455 (1.911-6.245) | <0.0001 |
| cT stage | 1.625 (1.295-2.039) | <0.0001 |  | 2.547 (1.894-3.426) | <0.0001 |  | 2.530 (2.034-3.147) | <0.0001 |
| cN stage | 1.613 (1.136-2.291) | 0.007 |  | 2.284 (1.621-3.216) | <0.0001 |  | 3.343 (2.490-4.488) | <0.0001 |
|  |  |  |  |  |  |  |  |  |
| **External validation cohort** |  |  |  |  |  |  |  |  |
| **Rad-score** | **19.456 (8.245-45.91)** | **<0.0001** |  | **44.283 (19.50-100.6)** | **<0.0001** |  | **86.61 (41.28-181.71)** | **<0.0001** |
| Age(years) (≥60 vs. <60) | 1.280 (0.857-1.913) | 0.228 |  | 1.283 (0.872-1.887) | 0.206 |  | 1.005 (0.742-1.361) | 0.973 |
| Gender (male vs. female) | 1.002 (0.651-1.540) | 0.994 |  | 0.879 (0.585-1.320) | 0.534 |  | 0.786 (0.574-1.076) | 0.133 |
| Tumor size | 1.595 (1.071-2.376) | 0.022 |  | 3.067 (2.047-4.595) | <0.0001 |  | 4.641 (3.357-6.417) | <0.0001 |
| Differentiation | 2.322 (1.708-3.156) | <0.0001 |  | 2.625 (1.927-3.578) | <0.0001 |  | 3.016 (2.380-3.822) | <0.0001 |
| CEA | 1.238 (0.663-2.313) | 0.503 |  | 3.138 (1.885-5.223) | <0.0001 |  | 3.344 (2.181-5.126) | <0.0001 |
| CA199 | 5.749 (2.990-11.055) | <0.0001 |  | 5.549 (2.920-10.546) | <0.0001 |  | 7.266 (4.131-12.778) | <0.0001 |
| cT stage | 1.434 (1.261-1.631) | <0.0001 |  | 1.674 (1.473-1.903) | <0.0001 |  | 1.999 (1.794-2.227) | <0.0001 |
| cN stage | 1.656 (1.319-2.078) | <0.0001 |  | 2.148 (1.735-2.658) | <0.0001 |  | 4.004 (3.305-4.850) | <0.0001 |

| **Table S8**. Predictive accuracy of newly developed nomogram, Rad-score and clinicopathological characteristics. | | | |
| --- | --- | --- | --- |
| **Variables** | **AUC (95% CI)** | | |
|  | **pN1 *vs*. pN0** | **pN2 *vs*. pN0** | **pN3 *vs*. pN0** |
| **Training cohort** |  |  |  |
| Nomogram | 0.802(0.725-0.880) | 0.892(0.840-0.945) | 0.949(0.918-0.980) |
| Rad-score | 0.665(0.572-0.759) | 0.772(0.696-0.847) | 0.837(0.775-0.899) |
| cN stage | 0.657(0.562-0.752) | 0.670(0.582-0.757) | 0.776(0.710-0.843) |
| cT stage | 0.668(0.574-0.762) | 0.665(0.577-0.754) | 0.757(0.684-0.830) |
| Differentiation | 0.626(0.531-0.722) | 0.662(0.573-0.751) | 0.654(0.570-0.739) |
| CA199 | 0.601(0.500-0.701) | 0.623(0.531-0.714) | 0.651(0.572-0.730) |
|  |  |  |  |
| **Internal validation cohort** |  |  |  |
| Nomogram | 0.772(0.699-0.758) | 0.873(0.818-0.928) | 0.913(0.879-0.948) |
| Rad-score | 0.667(0.577-0.758) | 0.715(0.627-0.803) | 0.772(0.714-0.830) |
| cN stage | 0.646(0.558-0.734) | 0.718(0.634-0.802) | 0.788(0.732-0.844) |
| cT stage | 0.703(0.623-0.783) | 0.809(0.745-0.873) | 0.807(0.754-0.860) |
| Differentiation | 0.599(0.512-0.686) | 0.678(0.595-0.761) | 0.666(0.599-0.733) |
| CA199 | 0.607(0.513-0.702) | 0.582(0.486-0.678) | 0.619(0.551-0.688) |
|  |  |  |  |
| **External validation cohort** |  |  |  |
| Nomogram | 0.822(0.783-0.860) | 0.866(0.833-0.899) | 0.936(0.919-0.953) |
| Rad-score | 0.676(0.625-0.727) | 0.721(0.674-0.769) | 0.781(0.748-0.814) |
| cN stage | 0.675(0.623-0.728) | 0.698(0.647-0.749) | 0.816(0.784-0.848) |
| cT stage | 0.661(0.610-0.713) | 0.705(0.657-0.754) | 0.778(0.742-0.813) |
| Differentiation | 0.646(0.594-0.697) | 0.661(0.612-0.710) | 0.679(0.639-0.719) |
| CA199 | 0.587(0.527-0.646) | 0.584(0.527-0.640) | 0.608(0.567-0.649) |

pN: pathological N stage; Rad-score: Radiomics score.

| **Table S9**. The AUC of the nomogram, Rad-score and clinicopathological characteristics for pN1 *vs* pN2, pN1 *vs* pN3, pN2 *vs* pN3. | | | |
| --- | --- | --- | --- |
| **Variables** | **AUC (95% CI)** | | |
|  | **pN2 *vs*. pN1** | **pN3 *vs*. pN1** | **pN3 *vs*. pN2** |
| **Training cohort** |  |  |  |
| Nomogram | 0.596(0.496-0.695) | 0.752(0.673-0.832) | 0.683(0.603-0.763) |
| Rad-score | 0.634(0.537-0.730) | 0.719(0.634-0.805) | 0.566(0.478-0.654) |
| cN stage | 0.524(0.424-0.623) | 0.660(0.577-0.743) | 0.631(0.550-0.712) |
| cT stage | 0.511(0.409-0.612) | 0.598(0.506-0.691) | 0.614(0.531-0.696) |
| Differentiation | 0.541(0.441-0.642) | 0.533(0.439-0.626) | 0.508(0.423-0.594) |
| CA199 | 0.522(0.422-0.622) | 0.551(0.460-0.642) | 0.529(0.444-0.614) |
|  |  |  |  |
| **Internal validation cohort** |  |  |  |
| Nomogram | 0.664(0.559-0.769) | 0.750(0.673-0.827) | 0.612(0.524-0.701) |
| Rad-score | 0.537(0.425-0.649) | 0.587(0.491-0.684) | 0.557(0.462-0.651) |
| cN stage | 0.603(0.495-0.712) | 0.710(0.636-0.785) | 0.616(0.530-0.702) |
| cT stage | 0.633(0.526-0.741) | 0.635(0.541-0.728) | 0.506(0.414-0.597) |
| Differentiation | 0.596(0.486-0.705) | 0.580(0.488-0.673) | 0.516(0.423-0.608) |
| CA199 | 0.525(0.414-0.636) | 0.512(0.420-0.604) | 0.537(0.445-0.629) |
|  |  |  |  |
| **External validation cohort** |  |  |  |
| Nomogram | 0.627(0.564-0.690) | 0.796(0.757-0.836) | 0.688(0.641-0.735) |
| Rad-score | 0.558(0.493-0.623) | 0.632(0.580-0.684) | 0.571(0.518-0.624) |
| cN stage | 0.688(0.643-0.732) | 0.748(0.708-0.788) | 0.581(0.516-0.646) |
| cT stage | 0.564(0.509-0.619) | 0.642(0.587-0.698) | 0.568(0.503-0.633) |
| Differentiation | 0.528(0.475-0.582) | 0.545(0.489-0.602) | 0.518(0.452-0.583) |
| CA199 | 0.524(0.472-0.577) | 0.521(0.466-0.576) | 0.503(0.437-0.569) |

pN: pathological N stage; Rad-score: Radiomics score.

| **Table S10. List of radiomics features derived.** | | | | | | | | | |
| --- | --- | --- | --- | --- | --- | --- | --- | --- | --- |
| **Co-occurrence matrix (220)** | | | | | | | | | |
| S(1,0)AngScMom | S(2,0)AngScMom | | S(3,0)AngScMom | | | S(4,0)AngScMom | | S(5,0)AngScMom | |
| S(1,0)Contrast | S(2,0)Contrast | | S(3,0)Contrast | | | S(4,0)Contrast | | S(5,0)Contrast | |
| S(1,0)Correlat | S(2,0)Correlat | | S(3,0)Correlat | | | S(4,0)Correlat | | S(5,0)Correlat | |
| S(1,0)SumOfSqs | S(2,0)SumOfSqs | | S(3,0)SumOfSqs | | | S(4,0)SumOfSqs | | S(5,0)SumOfSqs | |
| S(1,0)InvDfMom | S(2,0)InvDfMom | | S(3,0)InvDfMom | | | S(4,0)InvDfMom | | S(5,0)InvDfMom | |
| S(1,0)SumAverg | S(2,0)SumAverg | | S(3,0)SumAverg | | | S(4,0)SumAverg | | S(5,0)SumAverg | |
| S(1,0)SumVarnc | S(2,0)SumVarnc | | S(3,0)SumVarnc | | | S(4,0)SumVarnc | | S(5,0)SumVarnc | |
| S(1,0)SumEntrp | S(2,0)SumEntrp | | S(3,0)SumEntrp | | | S(4,0)SumEntrp | | S(5,0)SumEntrp | |
| S(1,0)Entropy | S(2,0)Entropy | | S(3,0)Entropy | | | S(4,0)Entropy | | S(5,0)Entropy | |
| S(1,0)DifVarnc | S(2,0)DifVarnc | | S(3,0)DifVarnc | | | S(4,0)DifVarnc | | S(5,0)DifVarnc | |
| S(1,0)DifEntrp | S(2,0)DifEntrp | | S(3,0)DifEntrp | | | S(4,0)DifEntrp | | S(5,0)DifEntrp | |
| S(0,1)AngScMom | S(0,2)AngScMom | | S(0,3)AngScMom | | | S(0,4)AngScMom | | S(0,5)AngScMom | |
| S(0,1)Contrast | S(0,2)Contrast | | S(0,3)Contrast | | | S(0,4)Contrast | | S(0,5)Contrast | |
| S(0,1)Correlat | S(0,2)Correlat | | S(0,3)Correlat | | | S(0,4)Correlat | | S(0,5)Correlat | |
| S(0,1)SumOfSqs | S(0,2)SumOfSqs | | S(0,3)SumOfSqs | | | S(0,4)SumOfSqs | | S(0,5)SumOfSqs | |
| S(0,1)InvDfMom | S(0,2)InvDfMom | | S(0,3)InvDfMom | | | S(0,4)InvDfMom | | S(0,5)InvDfMom | |
| S(0,1)SumAverg | S(0,2)SumAverg | | S(0,3)SumAverg | | | S(0,4)SumAverg | | S(0,5)SumAverg | |
| S(0,1)SumVarnc | S(0,2)SumVarnc | | S(0,3)SumVarnc | | | S(0,4)SumVarnc | | S(0,5)SumVarnc | |
| S(0,1)SumEntrp | S(0,2)SumEntrp | | S(0,3)SumEntrp | | | S(0,4)SumEntrp | | S(0,5)SumEntrp | |
| S(0,1)Entropy | S(0,2)Entropy | | S(0,3)Entropy | | | S(0,4)Entropy | | S(0,5)Entropy | |
| S(0,1)DifVarnc | S(0,2)DifVarnc | | S(0,3)DifVarnc | | | S(0,4)DifVarnc | | S(0,5)DifVarnc | |
| S(0,1)DifEntrp | S(0,2)DifEntrp | | S(0,3)DifEntrp | | | S(0,4)DifEntrp | | S(0,5)DifEntrp | |
| S(1,1)AngScMom | S(2,2)AngScMom | | S(3,3)AngScMom | | | S(4,4)AngScMom | | S(5,5)AngScMom | |
| S(1,1)Contrast | S(2,2)Contrast | | S(3,3)Contrast | | | S(4,4)Contrast | | S(5,5)Contrast | |
| S(1,1)Correlat | S(2,2)Correlat | | S(3,3)Correlat | | | S(4,4)Correlat | | S(5,5)Correlat | |
| S(1,1)SumOfSqs | S(2,2)SumOfSqs | | S(3,3)SumOfSqs | | | S(4,4)SumOfSqs | | S(5,5)SumOfSqs | |
| S(1,1)InvDfMom | S(2,2)InvDfMom | | S(3,3)InvDfMom | | | S(4,4)InvDfMom | | S(5,5)InvDfMom | |
| S(1,1)SumAverg | S(2,2)SumAverg | | S(3,3)SumAverg | | | S(4,4)SumAverg | | S(5,5)SumAverg | |
| S(1,1)SumVarnc | S(2,2)SumVarnc | | S(3,3)SumVarnc | | | S(4,4)SumVarnc | | S(5,5)SumVarnc | |
| S(1,1)SumEntrp | S(2,2)SumEntrp | | S(3,3)SumEntrp | | | S(4,4)SumEntrp | | S(5,5)SumEntrp | |
| S(1,1)Entropy | S(2,2)Entropy | | S(3,3)Entropy | | | S(4,4)Entropy | | S(5,5)Entropy | |
| S(1,1)DifVarnc | S(2,2)DifVarnc | | S(3,3)DifVarnc | | | S(4,4)DifVarnc | | S(5,5)DifVarnc | |
| S(1,1)DifEntrp | S(2,2)DifEntrp | | S(3,3)DifEntrp | | | S(4,4)DifEntrp | | S(5,5)DifEntrp | |
| S(1,-1)AngScMom | S(2,-2)AngScMom | | S(3,-3)AngScMom | | | S(4,-4)AngScMom | | S(5,-5)AngScMom | |
| S(1,-1)Contrast | S(2,-2)Contrast | | S(3,-3)Contrast | | | S(4,-4)Contrast | | S(5,-5)Contrast | |
| S(1,-1)Correlat | S(2,-2)Correlat | | S(3,-3)Correlat | | | S(4,-4)Correlat | | S(5,-5)Correlat | |
| S(1,-1)SumOfSqs | S(2,-2)SumOfSqs | | S(3,-3)SumOfSqs | | | S(4,-4)SumOfSqs | | S(5,-5)SumOfSqs | |
| S(1,-1)InvDfMom | S(2,-2)InvDfMom | | S(3,-3)InvDfMom | | | S(4,-4)InvDfMom | | S(5,-5)InvDfMom | |
| S(1,-1)SumAverg | S(2,-2)SumAverg | | S(3,-3)SumAverg | | | S(4,-4)SumAverg | | S(5,-5)SumAverg | |
| S(1,-1)SumVarnc | S(2,-2)SumVarnc | | S(3,-3)SumVarnc | | | S(4,-4)SumVarnc | | S(5,-5)SumVarnc | |
| S(1,-1)SumEntrp | S(2,-2)SumEntrp | | S(3,-3)SumEntrp | | | S(4,-4)SumEntrp | | S(5,-5)SumEntrp | |
| S(1,-1)Entropy | S(2,-2)Entropy | | S(3,-3)Entropy | | | S(4,-4)Entropy | | S(5,-5)Entropy | |
| S(1,-1)DifVarnc | S(2,-2)DifVarnc | | S(3,-3)DifVarnc | | | S(4,-4)DifVarnc | | S(5,-5)DifVarnc | |
| S(1,-1)DifEntrp | S(2,-2)DifEntrp | | S(3,-3)DifEntrp | | | S(4,-4)DifEntrp | | S(5,-5)DifEntrp | |
|  |  | |  | | |  | |  | |
| **Histogram features (9)** | **Run-length matrix (20)** | | | | | **Wavelet transform (20)** | | | |
| Mean | Horzl_RLNonUni | | 45dgr_RLNonUni | | | WavEnLL_s-1 | | | WavEnLL_s-4 |
| Variance | Horzl_GLevNonU | | 45dgr_GLevNonU | | | WavEnLH_s-1 | | | WavEnLH_s-4 |
| Skewness | Horzl_LngREmph | | 45dgr_LngREmph | | | WavEnHL_s-1 | | | WavEnHL_s-4 |
| Kurtosis | Horzl_ShrtREmp | | 45dgr_ShrtREmp | | | WavEnHH_s-1 | | | WavEnHH_s-4 |
| Perc.01% | Horzl_Fraction | | 45dgr_Fraction | | | WavEnLL_s-2 | | | WavEnLL_s-5 |
| Perc.10% | Vertl_RLNonUni | | 135dr_RLNonUni | | | WavEnLH_s-2 | | | WavEnLH_s-5 |
| Perc.50% | Vertl_GLevNonU | | 135dr_GLevNonU | | | WavEnHL_s-2 | | | WavEnHL_s-5 |
| Perc.90% | Vertl_LngREmph | | 135dr_LngREmph | | | WavEnHH_s-2 | | | WavEnHH_s-5 |
| Perc.99% | Vertl_ShrtREmp | | 135dr_ShrtREmp | | | WavEnLL_s-3 | | |  |
|  | Vertl_Fraction | | 135dr_Fraction | | | WavEnLH_s-3 | | |  |
|  |  | | |  | | WavEnHL_s-3 | | |  |
|  |  | | |  | | WavEnHH_s-3 | | |  |
|  | |  | | |  | |  | |  |
| **Gradient-based histogram features (5)** | | | | | **Autoregressive model (5)** | | | |  |
| GrMean | |  | | | Teta1 | |  | |  |
| GrVariance | |  | | | Teta2 | |  | |  |
| GrSkewness | |  | | | Teta3 | |  | |  |
| GrKurtosis | |  | | | Teta4 | |  | |  |
| GrNonZeros | |  | | | Sigma | |  | |  |

Perc. 10% = 10% gray-level percentile; Perc.90% = 90% gray-level percentile; SumAverg = sum average; SumOfSqs = Sum of squares; DifEntrp = difference entropy; DifVarnc = difference variance;AngScMom = angular second moment energy; Correlat = correlation; SumEntrp = sum entropy; InvDfMom = inverse differencemoment; 45dgr RLNonUni = 45^◦^ run-length non-uniformity; 45dgr Fraction and LngREmph = 45^◦^ fraction of image in runs and long run emphasis; 45dgr GLevNonU = 45^◦^ gray-level non-uniformity; Horzl RLNonUni = horizontal run-length non-uniformity; GrKurtosis = gradient kurtosis;WavEnLH s-3 = energy of wavelet transform coefficients in subband LH, with subsampling factor s = 3.S(x, y) indicates parameters of the co-occurrence matrix computed for different directions and distances between imagepixels; parameters computed for 4 directions: (a, 0), (0, a), (a, a), (a, –a), and 5 distances: a = 1, 2, 3, 4, 5, between image pixels.

A total of 279 texture parameters for each sample grouped into 6 main categories (histogram features, autoregressive models, co-occurrence matrix, gradient features, run-length matrix, wavelet transform).
